# Reassignment of a rare sense codon to a non-canonical amino acid in *Escherichia coli*

**DOI:** 10.1093/nar/gkv787

**Published:** 2015-08-03

**Authors:** Takahito Mukai, Atsushi Yamaguchi, Kazumasa Ohtake, Mihoko Takahashi, Akiko Hayashi, Fumie Iraha, Satoshi Kira, Tatsuo Yanagisawa, Shigeyuki Yokoyama, Hiroko Hoshi, Takatsugu Kobayashi, Kensaku Sakamoto

**Affiliations:** 1 Division of Structural and Synthetic Biology, RIKEN Center for Life Science Technologies, 1-7-22 Suehiro-cho, Tsurumi, Yokohama 230-0045, Japan; 2 RIKEN Systems and Structural Biology Center, 1-7-22 Suehiro-cho, Tsurumi, Yokohama 230-0045, Japan; 3 RIKEN Structural Biology Laboratory, 1-7-22 Suehiro-cho, Tsurumi, Yokohama 230-0045, Japan

## Abstract

The immutability of the genetic code has been challenged with the successful reassignment of the UAG stop codon to non-natural amino acids in *Escherichia coli*. In the present study, we demonstrated the *in vivo* reassignment of the AGG sense codon from arginine to l-homoarginine. As the first step, we engineered a novel variant of the archaeal pyrrolysyl-tRNA synthetase (PylRS) able to recognize l-homoarginine and l-*N*^6^-(1-iminoethyl)lysine (l-NIL). When this PylRS variant or HarRS was expressed in *E. coli*, together with the AGG-reading tRNA^Pyl^_CCU_ molecule, these arginine analogs were efficiently incorporated into proteins in response to AGG. Next, some or all of the AGG codons in the essential genes were eliminated by their synonymous replacements with other arginine codons, whereas the majority of the AGG codons remained in the genome. The bacterial host's ability to translate AGG into arginine was then restricted in a temperature-dependent manner. The temperature sensitivity caused by this restriction was rescued by the translation of AGG to l-homoarginine or l-NIL. The assignment of AGG to l-homoarginine in the cells was confirmed by mass spectrometric analyses. The results showed the feasibility of breaking the degeneracy of sense codons to enhance the amino-acid diversity in the genetic code.

## INTRODUCTION

In the ‘universal’ genetic code, the 64 possible permutations of the base triplets are assigned to the 20 canonical amino acids and translation stops. This redundancy in the codon assignments provides an opportunity to include a novel repertoire of amino acids within the framework of the triplet-based code. The UAG codon, one of the three stop codons, has thus been reassigned to non-natural amino acids in *Escherichia coli*, by eliminating the UAG-recognizing release factor 1 (RF-1) from the cell (1–8). The specific pair of a UAG-reading tRNA and an aminoacyl-tRNA synthetase (aaRS) variant has been developed for each of more than 100 kinds of synthetic amino acids (9), and they are available for assigning UAG to the new meanings. The stop-codon reassignment has benefited from the fact that UAG is the rarest codon in *E. coli*, and occurs only about 300 times in the genome and only at the end of a gene. UAG can be safely redefined, with a significant portion of the UAG codons remaining at the ends of open reading frames (ORFs) (1–7). The translation of these remaining UAG codons adds extra peptides to the C-termini of the protein products, but does not change their in-frame sequences.

By contrast, a sense codon occurs thousands of times in the genome, and is part of the coding sequence of a gene. The redefinition of a sense codon would change the amino-acid sequences of many proteins at the same time, as originally noted by Crick (10). This difference makes the redefinition of a sense codon more challenging (11–15). Although missense suppression, involving sense codons, has been utilized to incorporate synthetic amino acids into proteins, in addition to the 20 canonical molecules (16–19), the original assignments of these codons are preserved to avoid a destructive effect on the proteome. It has been proposed that the lethality of sense codon reassignment could be circumvented, if the codon to be redefined is totally eliminated from the genome by synonymous substitutions or neutral mutations, prior to the reassignment event (20). However, this scenario, which assumes the directed accumulation of a large number of mutations in the genome, rather explains the invariance of the codon assignments in the ‘frozen’ genetic code, and disfavors the adjustment of the code.

In the present study, we identified the laboratory conditions required for safely redefining a sense codon in *E. coli*. The AGG codon, one of the six arginine codons, is the rarest sense codon in the bacteria genome. We eliminated AGG codons from the essential genes by synonymous replacements, leaving the majority of the AGG codons in the genome. Then, the tRNA system involved in the translation of AGA and AGG was modified, to allow the two codons to be separately translated. We showed that these two conditions, together with the development of the pair of an aaRS and tRNA for translating AGG to a synthetic amino acid, were sufficient for AGG redefinition.

## MATERIALS AND METHODS

### Non-canonical amino acids

l-Homoarginine and l-*N*^6^-(1-iminoethyl)lysine (l-NIL) were purchased from Bachem.

### Plasmid construction

The prACYC184 plasmid was a derivative of pACYC184 created by changing an AGA codon at position 203 in the *cat* gene to CGA. The SUMO tag was derived from pET-SUMO-HV1 (3). The plasmid pHar was derived from pPylF (2), by inserting the gene coding for HarRS, the isolated PylRS variant specific for l-homoarginine, two tandem copies of the tRNA^Pyl^_CCU_ gene each with the *lpp^P5^* promoter (21) and the *rrnC* terminator (22), and a gentamicin resistance gene (*gent*). AGA at position 16 in the *rop* gene in the replication origin was changed to CGA in pHar. The codon usage in the HarRS gene was optimized, and thus had no AGA and AGG codons. The *gent* gene had AGG, AGA, and CGG replaced with other arginine codons.

The pBeta plasmid was previously described, and carries the gene for the single-stranded DNA annealing protein Beta under the control of the *tac* promoter, together with the *lacI^q^* gene (3). The plasmid pBeta-RF1 was derived from pBeta, by inserting the ORF of the *prfA* gene, together with the Shine-Dalgarno sequence, downstream of the ampicillin marker gene (*bla*).

The *argU* gene sandwiched between the core part of the *tyrT* promoter (23) and the *rrnC* terminator (22) was cloned into pACYC184, to create the plasmid pKS3-*argU*. Similarly, the *argW* gene sandwiched between these sequences was cloned in prACYC184, to create prKS3-*argW*. The *argU* gene from pKS3-*argU* was cloned into a plasmid having the pSC101^ts^ origin from pSC101-BAD (Gene Bridges GmbH), to create pSC101^ts^-*argU*. The same *argU* gene system was cloned in pBeta-RF1, together with the *gba* operon from the BL21(DE3) chromosome placed under the control of the arabinose promoter system (from pBAD TOPO, Invitrogen), in place of the *lacI^q^* and *bet* to create pGBA-RF1-*argU*. The kanamycin marker was then removed from pGBA-RF1-*argU*, to create pGBA-Amp-RF1-*argU*.

The plasmid pLp105 was derived from pAp15 (3) with the following modifications. The *repZ* gene had CGG codons in place of AGG codons at positions 33, 43, 231, and CGA in place of AGA at position 261, with a TAG-to-TAA change at the end of the ORF. The *repY* gene had TTA in place of AGA at position 6. The kanamycin marker gene had CGC in place of AGA at position 71. The *ispU-cdsA-rseP*, *dnaE-accA*, and *rpmH-rnpA* operons from the chromosome of BW25113 (the National BioResource Project, Japan), together with the *prfB* gene from the BL21(DE3) chromosome, were cloned in pLp105 to create pAGG11. The operons were placed in the same direction in terms of transcription. In the resulting plasmid, the AGG codons in *ispU-cdsA-rseP* and *dnaE* were changed to CGA, and the AGG codons in *prfB* and *rnpA* were changed to CGG. This AGG-to-CGG change in *prfB* was accompanied by a change of CTTTGA to CTTGA in the programmed +1 frameshift window (CUUUGA) (24), which converted the frameshift-dependent *prfB* expression to a constitutive expression, as reported previously (5,15). The wild-type T4 tRNA^Arg^_UCU_ (25) was engineered to have A30-U40 and G31-C39 base pairs. The tRNA^T4^_UCU_ variant sequences sandwiched between the indicated promoters and the *rrnC* terminator, as shown in Supplementary Table S1, were each cloned upstream of the *ispU* gene in pAGG11.

### PylRS engineering

The Leu, Leu, Asn, Cys and Tyr residues at positions 305, 309, 346, 348 and 384, respectively, in the amino-acid binding pocket of pyrrolysyl-tRNA synthetase (PylRS) from *Methanosarcina mazei* were randomly mutated, to generate an initial library of PylRS variants. Variants were expressed together with tRNA^Pyl^, and examined for the ability to suppress an amber mutation in the chloramphenicol (Cm) acetyltransferase gene, as reported previously (26,27). After three rounds of selecting ‘positive’ clones and two negative-selection rounds, we isolated a variant with the L305H, L309W, N346D, C348S and Y384F substitutions, which conferred a resistance to 75 μg/ml Cm in the presence of 1 mM l-homoarginine in the growth medium, and did not confer a resistance to 25 μg/ml Cm in the absence of the amino acid. Five additional substitutions (R61K, H63Y, S193R, N203T, and one of G444E, K429M, and L367M as the fifth) were found to increase the Cm resistance up to 200 μg/ml. Further selection exploited the observation that efficient UAG translation facilitates the growth of RFzero cells (2). *E. coli* BW25113 RFzero-iy was then transformed with plasmids expressing PylRS variants and tRNA^Pyl^, and inoculated on LB agar plates containing l-homoarginine (1 or 5 mM). The largest colonies were kept for further rounds of selection, with additional random mutations incorporated into the selected clones at each round. The final variant, HarRS, contained the substitutions R61K, H63Y, S193R, N203T, L305H, L309W, N346D, C348S, L367M, Y384F, K429M, K431M, D433G and G444E. Most of these positions were subject to random mutation, and it was confirmed that these substitutions were those conferred the best Cm resistance.

### HarRS structure modeling

The structural models of HarRS docked with l-homoarginine were created, using the PylRS structures (PDB entries: 2ZCE and 2ZIM) as templates. l-Homoarginine was manually docked into the HarRS active site, using CueMol (http://cuemol.sourceforge.jp/en/) and PyMOL (The PyMOL Molecular Graphics System, Version 0.99, Schrödinger, LLC), and the model was subjected to ten rounds of structure idealization with REFMAC5 (http://www.ccp4.ac.uk/html/refmac5.html).

### *In vitro* aminoacylation assay

The preparation of aaRS and tRNA and the *in vitro* aminoacylation assays were performed as described previously (28,29). The N-terminally tagged HarRS was synthesized in BL21(DE3). The aminoacylation reaction (20 μl) was performed in a 100 mM sodium–HEPES [4-(2-hydroxyethyl)-1-piperazineethanesulfonic acid] buffer (pH 7.2) containing HarRS (6.6 μM), the T7 transcript of tRNA^Pyl^ (4.6 μM), 10 mM MgCl_2_, 2 mM ATP, 4 mM dithiothreitol (DTT), and one of l-homoarginine, l-NIL, arginine, and phenylalanine (10 mM each). The reaction mixture was incubated at 37°C for up to 2 h, and the products were analyzed by acidic urea polyacrylamide gel electrophoresis [(10% (w/v) polyacrylamide gel) (pH 5.0)] as described previously (28–30). The specific activities of HarRS with the amino-acid substrates (pmol aminoacyl-tRNA formed/pmol enzyme/min) (29) were calculated from the linear increase of the proportion of aminoacylated tRNA^Pyl^ molecules in the course of the 2-h incubation. The band intensity was analyzed by using the ImageJ software.

### *E. coli* strains and cell culturing

*E. coli* B-95.ΔA is a derivative of BL21(DE3) (3). The strain AGG-27.1 was created based on B-95.ΔA. The plasmid pBeta-RF1 was introduced into B-95.ΔA to allow iterative cycles of oligonucleotide-mediated recombination, followed by the analysis of the modified clones by colony-direct PCR (3,31). The mutagenic oligonucleotides used for engineering are listed in Supplementary Table S2. PCR primers are listed in Supplementary Table S3. The genomic modifications in AGG-27.1 were confirmed by genome resequencing, using MiSeq (Illumina). To create AGG-27.2, AGG-27.1 was transformed with pGBA-RF1-*argU*, and the *argW* and *argU* genes were then replaced in the chromosome by a spectinomycin resistance gene (*spec*) and a Zeocin resistance gene (*zeo*), respectively, each expressed from the EM7 promoter. pGBA-RF1-*argU* was finally replaced by pSC101^ts^-*argU*. For the knockouts of *argW* and *argU*, the left hand arm (lhm) (5′-AAAAACCCGGCATAAATGGCGAGGGTTTAAGCAATCGAGCGGCAGCGTA-3′) and the right hand arm (rhm) (5′-TCCTTGTGTTTATCCCTAAAACCACATAAAAACCGTAAATTAAATTCGAA-3′) were used for the *spec* insertion by recombination. Those of lhm and rhm for the *zeo* insertion were 5′-CAAGGGTTGACCGTATAATTCACGCGATTACACCGCATTGCGGTATCAAC-3′ and 5′-TTACAATTCAATCAGTTACGCCTTCTTTATATCCTCCATAATTCCAGAGT-3′, respectively. To create strain AGG-27.3, pSC101^ts^-*argU* harbored by this strain was substituted for by pGBA-Amp-RF1-*argU*, and then the *argW::spec* locus, together with the upstream FIS binding site, was replaced by the P*selC*-core-tRNA^T4^_UCU_-T*rrnC* sequence linked to the kanamycin (*kan*) marker from pLp105, by recombination using the lhm and rhm sequences (5′-AAAAACCCGGCATAAATGGCGAGGGTTTAAGCAATCGAGCGGCAGCGTAC-3′ and 5′-TCCTTGTGTTTATCCCTAAAACCACATAAAAACCGTAAATTAAATTCGAA-3′, respectively). Finally, pGBA-Amp-RF1-*argU* in the cells was replaced by the initial pSC101^ts^-*argU*. The AGG-27.2 strain was grown at 30°C, in a Luria-Bertani (LB) medium containing Zeocin (66 μg/ml), tetracycline (5 μg/ml), or spectinomycin (100 μg/ml). The AGG-27.3 strain was grown in an LB medium containing kanamycin (15 μg/ml) at 30°C. *E. coli* cells were transformed by using a Micro Pulser (Bio-Rad). The optical cell densities were measured with a Libra S11 Visible and UV spectrophotometer (Biochrom Ltd.). When indicated, a 0.5-M solution of l-homoarginine or l-NIL was supplemented in growth media at a final concentration of 5 mM.

### Complementation tests

Temperature-sensitive strains were transformed with the indicated plasmids. The transformants were pre-incubated overnight at 30°C, inoculated on LB agar plates, and incubated at 30°C or 42°C for 2 days. Complementation of the temperature-sensitivity was judged by the formation of colonies on LB agar plates incubated at 42°C after 2 days. Alternatively, 2 μl aliquots of serial dilutions (1:10) of overnight cultures were spotted from left to right on LB agar plates supplemented with the indicated arginine analogs, and cultured at 30°C or 42°C for 2 days.

### Protein synthesis and MALDI-TOF MS analysis

The SUMO protein C-terminally tagged with an AHHHHHH*L peptide (the asterisk indicates the AGG position) was expressed from the plasmid prACYC-SUMO-AGG under the control of T7 promoter in B-95.ΔA cells harboring pHar. The host cells were pre-cultured in LB growth medium containing 1% glucose. The pre-culture was mixed with 0.5 l of a growth media supplemented with l-homoarginine (5 mM) and no glucose in flasks. After an overnight incubation at 37°C with vigorous shaking, the cells were harvested by centrifugation. The tagged SUMO was purified on a Ni-Sepharose column using a 20 mM sodium-phosphate buffer (pH 7.4) containing 500 mM NaCl and imidazole (20 or 500 mM), and then dialyzed overnight. The purified protein was cleaved with the SUMO protease at 4°C overnight, and then mixed with a 20 μl aliquot of TALON Superflow Resin (GE Healthcare). After an incubation at room temperature for 30 min, the resin was placed into an empty column, and then washed three times each by a suspension in 0.8 ml of MilliQ-purified water and centrifugation at 1000 × g, and finally drained by centrifugation at 1000 × g. The peptides were eluted with a solution containing 5% acetonitrile and 0.5% trifluoroacetic acid (TFA), and desalted by using SPE C-TIP(C18) pipette tips (Nikkyo Technos, Co., Ltd). The desalted samples were mixed with the same amount of the CHCA/DHB (1:1) solution and then spotted on a thin layer of CHCA/acetone. The peptide samples were fragmented by collision-induced dissociation (CID) with air, and analyzed using an AB SCIEX TOF/TOF 5800 system in the reflector positive ion (2 kV) mode.

The tagged SUMO protein was expressed from prACYC-HIS-SUMO-AGG in AGG-27.3/Har, and thus additionally had an N-terminal hexahistidine sequence with no AGG position. The host cells harboring the plasmid were pre-cultured overnight in an LB growth medium containing 1% glucose and l-homoarginine. The culture was then mixed with 0.5 l of the growth medium only with the amino acid in flasks. After culturing for about 18.5 h at 37°C, 2.5 g of the cells were harvested by centrifugation. The tagged SUMO protein was purified on a Ni-Sepharose column, and the used buffers contained 20 mM sodium-phosphate (pH 7.4), 500 mM NaCl, and imidazole (10 or 500 mM). After the purification, the salinity was reduced to 108 mM NaCl by the HiTrap Q (GE Healthcare) binding buffer [20 mM Tris–HCl (pH 8.0) and 10 mM NaCl], and the sample was then applied to chromatography on a HiTrap Q column (GE Healthcare) with a salt gradient from 10 mM NaCl to 1 M NaCl in a 20 mM Tris–HCl (pH 8.0) buffer. The eluted proteins were further purified by successive chromatography on a Superdex 75 10/300 column (GE Healthcare) using a purification buffer containing 20 mM Tris–HCl (pH 8.0) and 300 mM NaCl and then on the HiTrap Q column. The purified tagged SUMO proteins were digested by the SUMO protease at 30°C overnight, and the C-terminal HHHHHH*L peptide was then purified using an Amicon Ultra 3k filter (Merck Millipore). For MALDI-TOF MS analyses, 100 μl of the peptide solution was mixed with 50 μl of a TFA solution (final 0.5%), and desalted using SPE C-TIP(C18) pipette tips. The desalted samples were analyzed by MALDI-TOF MS using CHCA as the matrix.

### Amino acid analysis

The soluble fractions of the total proteins were obtained from the B-95.ΔA, and AGG-27.3/Har cells cultured in LB media containing l-homoarginine (5 mM). The harvested cells were suspended in a phosphate-buffered saline solution, and then lysed by sonication. The insoluble fraction of the cells was removed by centrifugation. The proteins in the supernatant were then precipitated with trichloroacetic acid and washed with acetone. The washed sample was dissolved in a 70% formic acid solution and exsiccated, to be hydrolyzed and then exsiccated again. The sample was then dissolved in 0.02 N HCl by applying ultrasound for 10 or 20 min. After filtration through 0.45-μm pores, the sample was subjected to the analysis of amino-acid composition, which was commercially performed by Bio-Material Analysis, Research Resources Center, RIKEN Brain Science Institute (Wako, Japan).

## RESULTS

### The AGG arginine codon chosen as the reassignment target

Arginine is encoded by six codons, AGA, AGG, and CGN (N represents U, C, A and G). Among these codons, AGG is the rarest in *E. coli*, occurring 1400 times in about 1000 genes (15,32). In *E. coli* BL21(DE3), 32 of the AGG-containing genes are essential, with 38 AGG codons in them (Supplementary Table S4). This rarity of AGG in the *E. coli* genome makes AGG an appropriate target for reassignment. However, the decoding of AGG involves two minor tRNA^Arg^ species: tRNA^Arg4^, which is encoded by the *argU* gene and recognizes AGA and AGG with a UCU anticodon (33,34); and tRNA^Arg5^, which is encoded by the *argW* gene and recognizes AGG with a CCU anticodon. The wobble recognition of AGG by tRNA^Arg4^ is problematic, when the meaning of AGG is to be changed, separately from the meaning of AGA. The elimination of both tRNAs will prevent not only AGG, but also AGA, from being translated into arginine. Thus, we had to engineer a tRNA system to read these codons separately, as described later.

**Figure 1.**
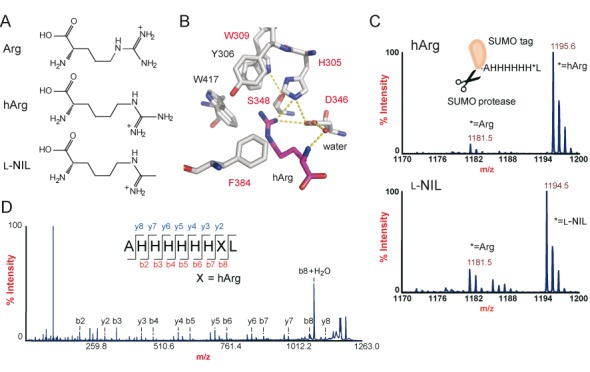
l-Homoarginine incorporation into proteins. (**A**) Chemical structures of arginine (Arg), l-homoarginine (hArg), and l-*N*^6^-(1-iminoethyl)lysine (l-NIL). (**B**) A docking model of the binding pocket of HarRS and hArg. hArg is in purple. The substituting amino acids are written in red. (**C**) MALDI-TOF MS analyses of the tag peptides, AHHHHHH*L (the asterisk indicates the AGG position), from the SUMO expressed in *E. coli* B-95.ΔA in the presence of hArg and l-NIL. The calculated *m/z* [M+H]^+^ values are 1181.6, 1195.6 and 1194.6 for the peptides containing arginine, hArg and l-NIL, respectively. (**D**) MS/MS spectrum of the l-homoarginine-containing peptide fragment. The *m/z* values for the peptide fragments are listed in Supplementary Table S5.

We conceived that the *in vivo* meaning of AGG would safely be changed to some arginine analogs, such as l-homoarginine, if AGG had been erased from some of the essential genes, prior to the event, by synonymous replacements with other arginine codons. The side chain of l-homoarginine is longer by one methylene, and more basic, than that of arginine (Figure 1A). This analog was expected to be generally compatible with arginine (35–38). However, despite the structural resemblance, l-homoarginine is not recognized by *E. coli* arginyl-tRNA synthetase (ArgRS) (39). Among the commercially available synthetic amino acids, l-*N*^6^-(1-iminoethyl)lysine (l-NIL) was another candidate as a safe substitute, due to its structural resemblance to l-homoarginine.

### Engineering a PylRS variant (HarRS) to attach arginine analogs to tRNA^Pyl^

To incorporate l-homoarginine into proteins, we modified the amino-acid specificity of PylRS, because this enzyme does not recognize any *E. coli* tRNA species as substrates, and its tRNA recognition mode does not involve the anticodon moiety (28). The PylRS―tRNA^Pyl^ pair has thus been used to translate sense codons to non-canonical amino acids (12,17,18). We isolated variants of *M. mazei* PylRS that can efficiently attach l-homoarginine to tRNA^Pyl^, from a library of variants harboring random mutations, some of which were located at the amino-acid binding pocket. Fourteen amino-acid substitutions occurred in the isolated variant designated as HarRS, with five of them (Leu305His, Leu309Trp, Asn346Asp, Cys346Ser and Tyr384Phe) being located within the amino-acid binding pocket. HarRS is the first PylRS variant reported as recognizing an amino acid with a strongly basic group in the side chain. A docking model of HarRS and l-homoarginine proposed that the guanidino group forms an electrostatic interaction with Asp346 and a hydrogen bond with His305 (Figure 1B and Supplementary Figure S1).

The acylation of tRNA^Pyl^ with l-homoarginine by HarRS was demonstrated by analyzing the products of *in vitro* aminoacylation reactions by acidic PAGE (Supplementary Figure S2). It was also shown that HarRS recognizes l-NIL less efficiently than l-homoarginine, whereas it does not recognize arginine. The acylation with phenylalanine was detected at a marginal level. The specific activities of HarRS with the amino-acid substrates, represented in pmol of the tRNA molecules acylated per pmol enzyme per min, were determined as 0.0015 and 0.0013 min^−1^ for l-homoarginine and l-NIL, respectively. These values are more than ten-times smaller than those reported for the wild-type PylRS towards pyrrolysine and *N*^ε^-(*tert*-butyloxycarbonyl)-l-lysine (0.04 and 0.09 min^−1^, respectively) under similar conditions (29).

### Incorporation of l-homoarginine and l-NIL by HarRS by missense suppression at the AGG codon

The anticodon of tRNA^Pyl^ was switched to CCU (Supplementary Figure S3), to translate AGG to the arginine analogs. A SUMO protein, C-terminally tagged with an Ala-His-His-His-His-His-His-*-Leu sequence (the asterisk indicates the AGG position), was expressed in B-95.ΔA, a BL21(DE3)-based *E. coli* strain, which was previously engineered to have no specific assignment for the UAG codon (3). The tag peptide was cleaved from the SUMO protein and analyzed by MALDI-TOF mass spectrometry, to identify the amino acids incorporated at the AGG position. When the pair of HarRS and tRNA^Pyl^_CCU_ were cloned in a plasmid, designated as pHar (Table 2), and co-expressed in the presence of l-homoarginine, the major peak corresponding to the peptide containing l-homoarginine was detected, together with the minor peak corresponding to the peptide with arginine (Figure 1C). An MS/MS analysis confirmed the incorporation of l-homoarginine at the AGG position (Figure 1D and Supplementary Table S5).

**Table 1.** *E. coli* strains used in this study

Strain	Genotype
BL21(DE3) (from Novagen)	F^−^*ompT gal dcm lon hsdS*_b_(*r*_b_^−^*m*_b_^−^) λ(DE3 [*lacI lacUV5*-T7 gene 1 *ind1 sam7 nin5*])
B-95.ΔA (3)	A Δ*prfA* derivative of BL21(DE3) with 95 UAG synonymous replacements, and 7 AGG synonymous replacements in *mrdB*, *ispE*, *asnS*, *pssA*, *ispB, mreC*, and *ligA*
AGG-27.1	A derivative of B-95.ΔA with 20 AGG replacements in *pheT*, *dnaC*, *yejM*, *lexA*, *coaE*, *trmD*, *hemG*, *ribF*, *asd*, *lpxC*, *folC*, *secE*, *yceQ*, *ftsL*, *ftsI*, *dnaG*, *lptF*, *ftsB*, and *glyQ* and 7 AGA replacements in *folC*, *trmD*, *dnaT*, *cca*, *lptC*, and *holB*
AGG-27.2	AGG-27.1 [*argU::zeo argW::spec*/ pSC101^ts^-*argU*]
AGG-38.2/Har^a^	AGG-27.2 [pAGG11-T3 pHar]
AGG-27.2/Har^a^	AGG-27.2 [pT3 pHar]
AGG-27.3	AGG-27.1 [*argU::zeo argW*::P*selC*-core-tRNA^T4^_UCU_-T*rrnC-kan*/ pSC101^ts^-*argU*]
AGG-27.3/Har^a^	AGG-27.3 [pHar]

^a^The pSC101^ts^-*argU* plasmid was eliminated by exposing the parent strain to the restriction temperature.

**Table 2.** Plasmids used in this study

Plasmid	Origin	Cloned genes	Markers
prACYC184	p15A	*cat*(AGA-free)	cm, tet
prACYC-SUMO-AGG	prACYC184	*lacI*(AGA326CGT)-PT7-SUMO-AHHHHHH*L (the asterisk indicates the AGG position)	cm
prACYC-HIS-SUMO-AGG	prACYC184	*lacI*(AGA326CGT)-PT7-6×His-SUMO-AHHHHHH*L (* = AGG)	cm
pHar	pBR322, AGA-free	*gent*(AGG/AGA/CGG-free), 2×P*lpp*^*P5*^-tRNA^Pyl^_CCU_-T*rrnC*, P*glnS*’-HarRS-T*rrnC*	gent
pBeta-RF1	pSC101	*lacI*^*q*^, P*tac*-*bet*-T*rrnB*, *bla-prfA*	kan, amp
pKS3-*argU*	p15A	P*tyrT*-core-tRNA^Arg4^-T*rrnC*	cm
pSC101^ts^-*argU*	pSC101^ts^	P*tyrT*-core-tRNA^Arg4^-T*rrnC*	tet
prKS3-*argW*	prACYC184	P*tyrT*-core-tRNA^Arg5^-T*rrnC*	cm
pGBA-RF1-*argU*	pSC101	*araC*-P*araBAD*-*gba*-T*rrnB*, *bla-prfA*, P*tyrT*-core-tRNA^Arg4^-T*rrnC, kan, bla*	kan, amp
pGBA-Amp-RF1-*argU*	pSC101	*araC*-P*araBAD*-*gba*-T*rrnB*, *bla-prfA*, P*tyrT*-core-tRNA^Arg4^-T*rrnC, bla*	amp
pLp105	ColIb-P9	pAp15, *kan*(AGA-free), *repY*(AGA-free), *repZ*(AGA/AGG/TAG-free)	kan
pAGG11	pLp105	*ispU-cdsA-rseP, dnaE-accA, prfB, rpmH-rnpA* with AGG synonymous replacements	kan
pAGG11a	pAGG11	The same as pAGG11 except for the antibiotic marker	amp
pAGG11-T1	pAGG11	pAGG11 with P*tyrT*-core-tRNA^T4^_UCU_-T*rrnC*	kan
pAGG11-T2	pAGG11	pAGG11 with P*selC*-tRNA^T4^_UCU_-T*rrnC*	kan
pAGG11-T3	pAGG11	pAGG11 with P*selC*-core-tRNA^T4^_UCU_-T*rrnC*	kan
pT3	pLp105	P*selC*-core-tRNA^T4^_UCU_-T*rrnC*	kan

When l-NIL was supplemented in the growth medium in place of l-homoarginine, the peptide containing l-NIL was detected as the major peak, together with the arginine-containing peptide as a minor peak (Figure 1C). These findings showed that the HarRS―tRNA^Pyl^_CCU_ pair can incorporate l-homoarginine and l-NIL at AGG, well competing with the endogenous machinery to translate AGG to arginine.

### Synonymous replacements of AGA and AGG codons in essential genes

**Figure 2.**
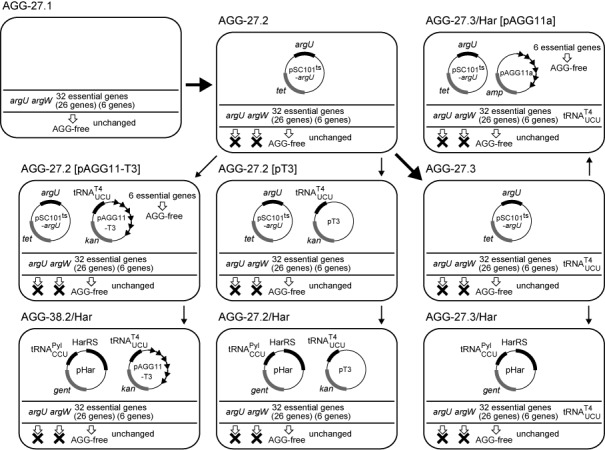
The genetic compositions of the representative *E. coli* strains used in this study. Their lineages are indicated by the arrows. The bold arrows indicate the modifications made by chromosomal engineering.

To safely change the meaning of the AGG codon, we tried to synonymously replace the 38 AGG codons in the 32 essential genes. Seven AGG codons in seven essential genes (*mrdB*, *ispE*, *asnS*, *pssA*, *ispB, mreC* and *ligA*) are already replaced in the starting strain B-95.ΔA (3) (Supplementary Table S6), by using the oligonucleotide-directed recombination method (31). Then, we applied the same technique and substituted CGN codons for 20 of the remaining 31 AGG codons, within 19 genes (*pheT*, *dnaC*, *yejM*, *lexA*, *coaE*, *trmD*, *hemG*, *ribF*, *asd*, *lpxC*, *folC*, *secE*, *yceQ*, *ftsL*, *ftsI*, *dnaG*, *lptF*, *ftsB* and *glyQ*), with two exceptional cases, where AGG could not be replaced with CGN, and was replaced with AGA or the AAG lysine codon (Supplementary Table S7). The CGN codons substituting for AGA or AGG were preferably CGC or CGT, which introduce mismatches favored by the employed method (31). Furthermore, seven AGA codons in six essential genes, *folC*, *trmD*, *dnaT*, *cca*, *lptC* and *holB*, were replaced synonymously in the chromosome (Supplementary Table S7), to create the strain AGG-27.1 (Figure 2). In each of these genes, the AGA codon occurred twice in a row or more than twice (Supplementary Table S4), and the concentration of such a rare codon in a gene can affect the gene expression (40). The purpose of replacing the seven AGA codons was to prevent the adverse effects possibly caused by a reduction in the availability of the AGA-reading tRNA in the final strain, as described later. The sequencing of the whole chromosome of strain AGG-27.1 confirmed the occurrence of all of the intended replacements, and detected the three off-target mutations listed in Supplementary Table S8.

The replacement of the final 11 AGG codons in the six genes, *ispU*, *cdsA*, *rseP*, *dnaE*, *prfB* and *rnpA* in the chromosome was not successful, probably because induced chromosomal base changes in essential ORFs are normally infrequent events, and our approach using a *mutS*^+^ background could not raise the mutational frequency with these genes. Then, these six essential genes were cloned in vector pLp105, which was a derivative of pAp102 (33) with a copy number of 1.7 per cell (41), and free of AGA, AGG and UAG codons. The 11 AGG codons in the genes were then replaced synonymously, to create pAGG11. The introduction of pAGG11 makes *E. coli* cells with the 27 AGG already being eliminated from the chromosome have the entire set of 32 AGG-free essential genes.

### Separate reading of AGA and AGG by two different tRNA species

Since tRNA^Arg4^ recognizes both AGA and AGG, the AGG-recognizing tRNA^Arg5^ species, encoded by *argW*, is dispensable in *E. coli* [Profiling of *E. coli* chromosome ver. 4 (http://www.shigen.nig.ac.jp/ecoli/pec/index.jsp)]. The cellular level of a tRNA species with a UCU anticodon might be reduced to the extent that this tRNA_UCU_ alone could not support the translation of both AGA and AGG, and it would thus be necessary for a tRNA_CCU_ species to assume the burden of AGG translation. In such a situation, AGG would be preferentially read by tRNA_CCU_, and could be allocated to synthetic amino acids, when tRNA^Pyl^_CCU_ was used.

This scenario requires the replacement of the endogenous tRNA^Arg4^ and tRNA^Arg5^ by artificial pairs of tRNA_UCU_ and tRNA_CCU_. Since it is lethal to remove the *argU* and *argW* genes at the same time, we engineered a derivative of AGG-27.1 expressing tRNA^Arg4^ from a plasmid that cannot be maintained at high temperature (pSC101^ts^-*argU*), while the *argU* and *argW* genes were knocked out in the chromosome. The resulting temperature-sensitive strain, AGG-27.2 (Figure 2 and Table 1), was used to identify a promoter to express tRNA_UCU_ at a desirable level. We chose a variant of the bacteriophage-T4 arginine tRNA_UCU_ with a stabilized anticodon stem (Supplementary Figure S3), as tRNA_UCU_, and expressed it from one of the following three promoters. The *tyrT* core promoter (*T1*) is a moderately strong promoter, while the *selC* promoter (*T2*) is the weakest *E. coli* promoter for tRNA (42), and it becomes even weaker when the promoter region is reduced to the core domain (*T3*) (43).

The tRNA^T4^_UCU_ genes with these promoters were cloned in the low-copy plasmid pAGG11, to create pAGG11-T1, and pAGG11-T2, and pAGG11-T3, respectively. Note that the AGG-27.2 cells transformed with one of these pAGG11 plasmids, each carrying the six genes lacking the 11 AGG codons, then had the entire set of 32 AGG-free essential genes. It was found that the temperature sensitivity of AGG-27.2 was not complemented by introducing pAGG11-T3, whereas pAGG11-T1 and pAGG11-T2 achieved the complementation (Supplementary Figure S4). This finding showed that the cellular level of tRNA^T4^_UCU_ expressed from the weakest promoter (*T3*) could not support the translation of both AGA and AGG, when the plasmid expressing tRNA^Arg4^ was eliminated from the cell at the high temperature. However, this expression level for tRNA^T4^_UCU_ was sufficient for the complementation, when tRNA^Arg5^ was co-expressed from another plasmid prKS3-*argW* (Table 2) (Supplementary Figure S5). Thus, in this situation, it is reasonably assumed that AGA and AGG are recognized preferentially by tRNA^T4^_UCU_ and tRNA^Arg5^, respectively, in the AGG-27.2 cells harboring pAGG11-T3.

### Reassignment of the AGG codon to l-homoarginine

**Figure 3.**
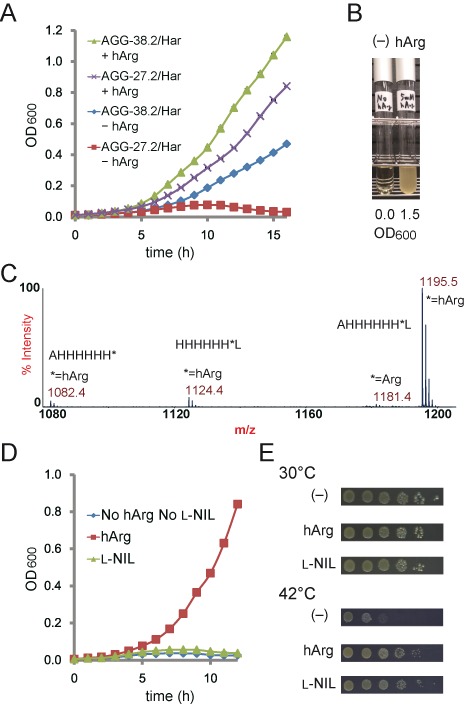
AGG codon reassignment. (**A**) Growth profiles of the AGG-38.2/Har and AGG-27.2/Har cells in the presence (+hArg) and absence (–hArg) of l-homoarginine in the growth media at 37°C with vigorous shaking. Each data point represents the average OD_600_ value of three independent experiments. The standard deviations were so small that errors bars are hardly visible in the graph. (**B**) Growth of the AGG-27.3/Har cells examined in the absence (left) and presence (right) of l-homoarginine supplemented in the growth media. The cells were incubated for 24 h at 37°C with vigorous shaking. (**C)** MALDI-TOF MS analysis of the peptide fragment AHHHHHH*L (the asterisk indicates the AGG position) expressed in AGG-27.3/Har. (**D**) Growth of the AGG-27.2/Har cells in the presence of hArg or l-NIL and their absence in the growth media at 37°C with vigorous shaking. Each data point represents the average OD_600_ value of three independent experiments. The standard deviations were so small that errors bars are hardly visible in the graph. (**E**) The overnight culture of the AGG-27.3/Har[pAGG11a] cells was serially diluted (1:10 at each step), spotted on LB agar plates containing the designated amino acids, and then incubated at 30 and 42°C for 2 days.

We assessed whether homoarginyl-tRNA^Pyl^_CCU_ can substitute for arginyl-tRNA^Arg5^ as the only tRNA species to translate AGG in the cell. The pair of HarRS and tRNA^Pyl^_CCU_ was expressed from the pHar plasmid. The temperature sensitivity of the AGG-27.2 cells with pAGG11-T3, which was shown earlier to be complemented by expressing tRNA^Arg5^, was also complemented by the introduction of pHar, together with the supplementation of l-homoarginine in the growth medium (Supplementary Figure S6). This observation indicated that the reassignment of AGG to l-homoarginine is tolerated, provided that the 38 essential AGG codons are synonymously replaced prior to the event. After the growth at the restrictive temperature, the *argU*-carrying pSC101^ts^ plasmid was absent in the cells, as shown by a loss of the tetracycline resistance conferred by the plasmid. Due to this change in the plasmid composition in the cells, we gave a different designation, AGG-38.2/Har (Figure 2 and Table 1), to the AGG-27.2[pAGG11-T3 pHar] cells that experienced exposure to the high temperature. This strain grew rapidly at 37°C, depending on the supplementation of l-homoarginine in the growth medium (Figure 3A). The observed slower growth even in the absence of this amino acid implied that tRNA^Pyl^_CCU_ was charged with arginine at a low level, when l-homoarginine was lacking, and then translated AGG into arginine; the mis-arginylation of another tRNA^Pyl^ variant with the CCG anticodon was previously reported (12).

We then examined if the AGG reassignment to l-homoarginine could be achieved only with the synonymous replacements of the 27 essential AGG codons in the chromosome. The six AGG-free genes were removed from pAGG11-T3 to create pT3, and AGG-27.2 was then transformed with this plasmid. The temperature-sensitivity of the AGG-27.2[pT3] transformant was complemented by the introduction of pHar in the presence of l-homoarginine (Supplementary Figure S6). This observation indicated that the AGG assignment can safely be changed to the arginine analog, with only 27 AGG codons eliminated from the essential genes. After exposure to high temperature to shed the *argU*-carrying pSC101^ts^ plasmid, the resulting AGG-27.2/Har cells (Figure 2 and Table 1) grew at 37°C with a strict dependence on the supplementation of l-homoarginine, but did not grow as fast as AGG-38.2/Har (Figure 3A). From this viability of AGG-27.2/Har, the six essential genes left with 11 AGG codons were thought to express the functional proteins containing l-homoarginines at the AGG positions in this strain. The locations and functions of the replaceable arginine residues in these proteins are discussed in the ‘Supplementary Discussion’. The strict dependence of the growth of AGG-27.2/Har on the supplementation of l-homoarginine indicated that the level of the mis-arginylation of tRNA^Pyl^_CCU_ in the absence of the amino acid was probably insufficient for supporting the expression of the six essential genes still containing 11 AGG codons.

### Identification of the amino acids incorporated at AGG in *E. coli* cells with the non-canonical assignment for AGG

We performed mass spectrometric analyses to identify the amino acids incorporated into proteins in response to AGG, to support our claim that the codon is successfully reassigned to l-homoarginine. For this purpose, the gene encoding tRNA^T4^_UCU_ was inserted into the chromosome, instead of expressing it from a plasmid, to avoid a possible change in the plasmid copy number (44). The low cellular level of tRNA^T4^_UCU_ was crucial for preventing the translation of AGG as arginine, and should be maintained. The strain AGG-27.3 (Figure 2 and Table 1) was created by incorporating the tRNA^T4^_UCU_ gene under the control of the *selC* core promoter into the *argW* locus in the chromosome of AGG-27.2. The temperature-sensitivity of AGG-27.3 was complemented by expressing either tRNA^Arg5^ (Supplementary Figure S7A) or the pair of HarRS and tRNA^Pyl^_CCU_ in the presence of l-homoarginine (Supplementary Figure S7B). After exposure to high temperature to shed the *argU*-expressing plasmid, the resulting AGG-27.3/Har strain grew well in the presence of l-homoarginine, with the culture reaching an optical density at 600 nm (OD_600_) of 1.5 in 24 h at 37°C, whereas this strain did not grow without l-homoarginine (Figure 3B).

As the amino acids incorporated at AGG in B-95.ΔA was analyzed, the SUMO was tagged with the peptide AHHHHHH*L (The asterisk indicates the AGG position), and was expressed in AGG-27.3/Har. MALDI-TOF MS analyses of the tag peptide fragment detected the major peak corresponding to the peptide with l-homoarginine at the AGG position (calculated *m/z* of 1195.6) (Figure 3C). The minor peaks at the *m/z* values of 1124.4 and 1082.4 corresponded to the homoarginine-containing peptides lacking the terminal alanine and leucine residues, respectively. The calculated *m/z* values for the Ala and Leu-lacking peptides were 1124.6 and 1082.6, respectively. By contrast, the peptide with arginine at AGG was detected at only a marginal level. These results strongly supported our claim that the AGG codon had been reassigned to l-homoarginine.

### Overall incorporation of l-homoarginine in the proteome of the AGG-redefined *E. coli* cells

We then tried to demonstrate that l-homoarginine was incorporated in the endogenous proteins of the AGG-27.3/Har cells, in which most of the AGG codons remained in the genome. The frequency of the occurrence of AGG in the *E. coli* genome is 2.0%, relative to the combined frequencies of the other arginine codons (45). Considering that most of the AGG codons are not contained in the highly expressed genes, the relative abundance of the AGG-encoded l-homoarginine in the proteins is expected to be much smaller than the relative frequency in the genes. l-Homoarginine and arginine were separately detected in an analytical chromatography, with the retention times of 113 min and 100 min, respectively. Only the soluble protein fraction was analyzed for the convenience of experiments. Thus, the proteins from B-95.ΔA and AGG-27.3/Har were found to contain l-homoarginine in the relative amounts of 0.0% and 0.4%, respectively, as compared with the abundance of arginine (Supplementary Figures S8 and S9). Since B-95.ΔA was cultured in a medium containing l-homoarginine and showed no fraction of this amino acid in the proteins, we could exclude the possibility of the contamination of the analyzed samples by l-homoarginine from the media. Thus, the observed fraction of l-homoarginine indicated the incorporation of the arginine analog into the proteome of the AGG-redefined *E. coli* cells.

### AGG reassignment with l-NIL

HarRS can also recognize l-NIL as a substrate, as shown previously. The temperature sensitivity of the AGG-27.2[pAGG11-T3 pHar] cells, which expressed tRNA^T4^_UCU_ and the HarRS―tRNA^Pyl^_CCU_ pair, was complemented when l-NIL was substituted for l-homoarginine in the growth medium, although the activity of complementation was slightly weaker for l-NIL than l-homoarginine (Supplementary Figure S10). A similar result was obtained by using the AGG-27.3/Har cells transformed with pAGG11a, the same plasmid as pAGG11 except for the antibiotic marker (Figure 3E). Both strains had the entire set of the 32 essential genes free of AGG. These observations suggested that AGG can safely be reassigned to l-NIL, which lacks a guanidino group, unlike arginine and l-homoarginine, provided that all 38 of the essential AGG codons are replaced synonymously. However, the poor growth of these cells with AGG reassigned to l-NIL and poor amounts of the tagged SUMO proteins prevented mass spectrometric analyses to identify the amino acid translated from the AGG codon.

By contrast, the translation of AGG to l-NIL did not support cell growth, when only the 27 essential genes were replaced. The AGG-27.2[pT3 pHar] cells did not show the complementation activity (Supplementary Figure S10). The AGG-27.2/Har cells pre-cultured with l-homoarginine did not grow in a medium containing l-NIL, whereas these cells grew well with l-homoarginine (Figure 3D). In these strains, the six essential genes with 11 AGG codons remained unchanged. The observed inability of AGG-27.2/Har to grow in the presence of l-NIL suggested that the gene products of the six essential genes, which were shown functional with l-homoarginine at the AGG positions, were not functional with l-NIL at the same positions.

## DISCUSSION

In the present study, we succeeded to unambiguously redefine the AGG codon with l-homoarginine, by developing HarRS, which efficiently acylates tRNA^Pyl^_CCU_ with the arginine analog, and eliminating the redefined codon from the essential genes. Engineering a tRNA system to translate AGA and AGG separately allowed us to address only AGG, the rarest sense codon in the *E. coli* genome. The results of complementation tests suggested that AGG was probably reassigned to l-NIL. The barrier to the reassignment to l-NIL was reasonably higher than that to l-homoarginine, with the higher degree of the dissimilarity between l-NIL and arginine. The observation that the *E. coli* cells with redefined AGG grew with less vigor than the wild-type cells indicated that the substitution of these arginine analogs for arginine in ‘non-essential’ proteins can have adverse effects on the cell's viability. Further replacements of AGG, not only in the essential genes, would facilitate the redefinition of the codon with a more variety of amino acids, as shown in the case of the UAG stop codon (2,3,8).

Sense codon reassignment involving the AGA, AGG and CGG arginine codons was previously attempted, with no established success (12,18). With the current technology, it is not easy to totally eliminate these codons from the genome, although they occur with the least frequencies in the *E. coli* genes. If it is feasible, our knowledge of the effects of such a large-scale genomic engineering involving a sense codon is incomplete (15). AGG was previously reassigned to synthetic amino acids ambiguously by missense suppression, with the endogenous activity to translate AGG being retained in *E. coli* (18,19). In *Mycoplasma capricolum*, a CGG-reading tRNA^Pyl^ variant was used to encode a synthetic amino acid (12), because there are few CGG codons in the genome (46). However, the endogenous ArgRS more efficiently acylated the tRNA^Pyl^ variant than PylRS, resulting in the assignment of CGG to arginine. A similar reassignment of a sense codon to a canonical amino acid was reported for *Candida albicans* (47). A recent evolutionary engineering successfully substituted a synthetic amino acid for tryptophan in the *E. coli* proteome, although this total substitution for tryptophan did not increase the number of different amino acids concurrently included in the genetic code (48).

It has been proposed that the degeneracy in the codon assignments was continuously reduced, with the inclusion of ‘new’ amino acids in the genetic code, until the modern code was established (10,49,50). This might be the case for glutamine and asparagine. All of the CAA, CAG, GAA and GAG codons possibly once specified glutamate, with the first two having later changed their meaning to encode glutamine (49). Similarly, the modern asparagine codons, AAU and AAC, might have once encoded aspartate, together with the invariant GAU and GAC aspartate codons. Tryptophan and methionine are unique, in that each of them is encoded by a single codon, and thus they are thought to be the newest additions to the genetic code (50). Once the modern nuclear code was established, the amino-acid repertoire has never changed, and the assignments to the codons have been ‘frozen’ (10), except for minor deviations found in certain organisms (49). However, recent advances in biotechnology have allowed the redefinition of one of the three stop codons with designer amino acids *in vivo*, demonstrating the feasibility of reducing the codon degeneracy to expand the repertoire of genetically encoded amino acids (1–8). By contrast, degenerate sense codons are found in almost every corner of the code, and could be exploited to incorporate multiple new amino acids into proteins. Although the present AGG reassignment was confined to a close analogue of arginine, the present results suggested that the sense codons are not hard-wired to their original meanings in the standard code.

A number of *in vivo* deviations concerning the meanings of sense codons have been discovered in the mitochondrial genomes (51). Although the occurrence of such deviations is largely facilitated by the small size of the mitochondrial genome, a change in the translation machinery was reportedly involved in the deviation of a codon meaning. In the invertebrate mitochondria, the six ‘universal’ arginine codons are split into CGN for arginine and AGA/AGG for serine (51). The mitochondrial ArgRS does not mis-acylate tRNA^Ser^_UCU_, because it has a tRNA recognition mode slightly different from that of the nuclear ArgRS, and this altered recognition enables distinguishing between tRNA^Ser^_UCU_ and tRNA^Arg^_UCG_ (52). This case illuminates the importance of an adjustment in the endogenous tRNA―aaRS system for codon redefinition, as has been proposed (53,54) and shown with our engineering of a new tRNA system.

Engineered genetic codes could confer selective advantages on organisms. A T4-bacteriophage variant, with better ability to propagate among the host cells, reportedly evolved in the *E. coli* strain with UAG reassigned to 3-iodo-l-tyrosine (55). Spontaneous mutations created in-frame UAG codons, and 3-iodotyrosine was thus incorporated into some of the T4 proteins. Although the advantages that the incorporated 3-iodotyrosines conferred on the bacteriophage were not elucidated, a recent report showed that the incorporation of 3-halogenated tyrosine derivatives can enhance the structural stability of proteins (56). l-Homoarginine was substituted for arginine at most of the AGG positions in the *E. coli* proteome in the present study. If the effect of a particular substitution is not favorable for the cell, then the AGG codon could be mutated to CGG or AGA, to restore arginine at the position. If the effect is favorable or neutral, then the AGG codon would remain unchanged. Arginine and lysine residues might be replaced with l-homoarginine, by single-base mutations to change AGA/CGG to AGG, and AAG to AGG, respectively. Thus, sense codon reassignments provide a different and more extensive laboratory model for the evolution of a new proteome, as compared to the model provided by stop-codon reassignments, because of the higher frequency of sense codon occurrence in the genome.

## Supplementary Material

SUPPLEMENTARY DATA
