# Supplementary material for: Reassignment of a rare sense codon to a non-canonical amino acid in Escherichia coli
Source: Nucleic Acids Res. 2015 Aug 3;43(16):8111–22. doi: 10.1093/nar/gkv787 (PMC4652775; doi:10.1093/nar/gkv787)
Supplement: SUPPLEMENTARY DATA [file supp_43_16_8111__index.html]

Reassignment of a rare sense codon to a non-canonical amino acid in Escherichia coli — SUPPLEMENTARY DATA 

# Reassignment of a rare sense codon to a non-canonical amino acid in *Escherichia coli*

## SUPPLEMENTARY DATA

- SUPPLEMENTARY DATA
