# Supplementary material for: Reassignment of a rare sense codon to a non-canonical amino acid in Escherichia coli
Source: Nucleic Acids Res. 2015 Aug 3;43(16):8111–22. doi: 10.1093/nar/gkv787 (PMC4652775; doi:10.1093/nar/gkv787)
Supplement: SUPPLEMENTARY DATA [file supp_gkv787_Supplementary_Mukai_correction.pdf]

Reassignment of a rare sense codon to a non-canonical amino acid in *Escherichia coli*

Takahito Mukai, Atsushi Yamaguchi, Kazumasa Ohtake, Mihoko Takahashi, Akiko Hayashi, Fumie Iraha, Satoshi Kira, Tatsuo Yanagisawa, Shigeyuki Yokoyama, Hiroko Hoshi, Takatsugu Kobayashi and Kensaku Sakamoto

This PDF file includes:
Supplementary Discussion
Supplementary Figures S1-S10
Supplementary Tables S1-S8

SUPPLEMENTARY DISCUSSION

The safe arginine-to-homoarginine replacements at the 11 AGG positions in the 6 essential genes may be explained on the basis of their reported protein structures. Among the 11 arginine residues, only Arg283 in CdsA is not conserved among the orthologs and may be replaceable with other amino acids. The other 10 arginine residues are completely conserved among the *Enterobacteriaceae* family members, including *E. coli*. Arg156 in IspU is bound to the main chain of Asp170 and Ile172 in the other subunit (PDB ID: 3QAS) and may be replaceable with L-homoarginine, because this arginine residue is replaced by lysine in some of the eukaryotic orthologs. Arg245 in RseP forms a salt bridge with Glu223 on the protein surface (PDB ID: 3ID2), and may be changed to L-homoarginine without destroying the salt bridge. Arg278 in RseP forms a salt bridge with Asp244 and a hydrogen bond with the main chain of Gly239, and is partially buried at the protein surface (PDB IDs: 3ID2). These interactions may be partially destabilized by the arginine-to-homoarginine replacement. Arg194 in IspU participates in a hydrogen bond network and is mostly buried in the protein core (PDB ID: 3QAS), and the hydrogen bond network may be restructured by the arginine-to-homoarginine replacement. Arg175 in DnaE recognizes the substrate PO_4^{3-} (PDB IDs: 2HNH and 2HQA), and may be changed to L-homoarginine without affecting the substrate specificity, because DnaE has sufficient space to accommodate the longer side chain of L-homoarginine. In DnaE, Arg981, Arg1091, and Arg1121 may be located around the interface with the subunits and the DNA substrate [Lamers, M. H., Georgescu, R. E., Lee, S. G., O'Donnell, M. and Kuriyan, J. (2006) Crystal structure of the catalytic alpha subunit of *E. coli* replicative DNA polymerase III. *Cell*, **126**, 881–892], and may be replaceable with L-homoarginine, because the interactions between large proteins and DNA would not be severely destabilized by such small changes. Arg8 in RnpA may form a long-distance contact with the sugar-phosphate backbone of the RNaseP RNA (PDB IDs: 2LJP and 3Q1R), and thus may be changed to L-homoarginine without hindering this interaction. Arg22 in RF-2 bridges two α-helixes and is located around the putative interface with RF-3 (PDB ID: 1GQE). RF2 may have sufficient space to accommodate the longer side chain of L-homoarginine without forming a protruding bulge. The observation that the wildtype proteins were more functional than the L-homoarginine-substituted variants suggests that the guanidino groups were moved from the original positions in some of these proteins. The observation that some of these 11 arginine residues cannot be replaced with L-NIL suggests that these groups were still essential for the functions of some of these proteins.

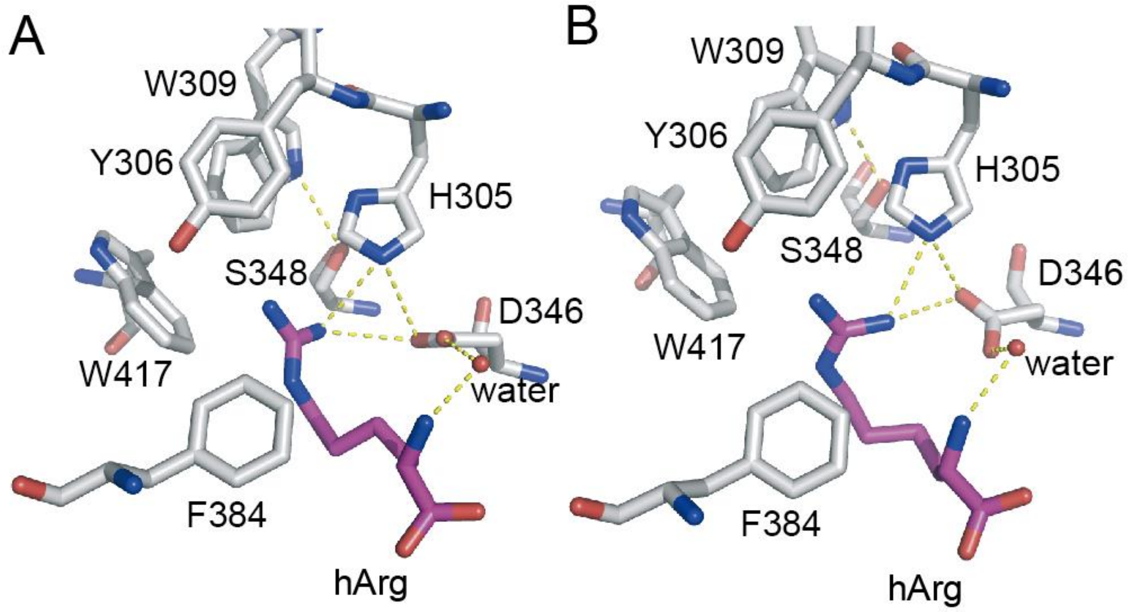

Figure S1. Structure models for the amino-acid binding pocket of the L-homoarginine-specific PylRS variant (HarRS). The two similar models (A, the same as Figure 1B, and B) were obtained with two different initial conditions for the manual docking of L-homoarginine. L-Homoarginine is represented by sticks, with the carbon atoms shown in purple.

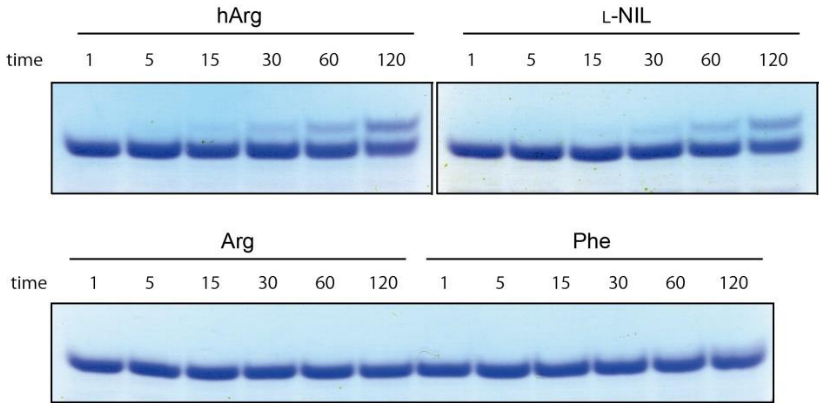

Figure S2. The acylation of $tRNA^{Pyl}$ with hArg, L-NIL, arginine, and phenylalanine by HarRS was analyzed by acid urea PAGE followed by the staining with toluidine blue. The reaction was performed at 37 °C for the indicated durations (min). The bands shifted upwards on the gel correspond to the acylated tRNA. The extent of the upward shift and the intensity of the shifted band were subtle for Phe. The proportion of the acylated molecules amounted to 26% and 22% for hArg and L-NIL, respectively, at the end of the 2-hr incubation. The increase in the acylated proportion was nearly linear for both amino acids over the indicated time range of incubation.

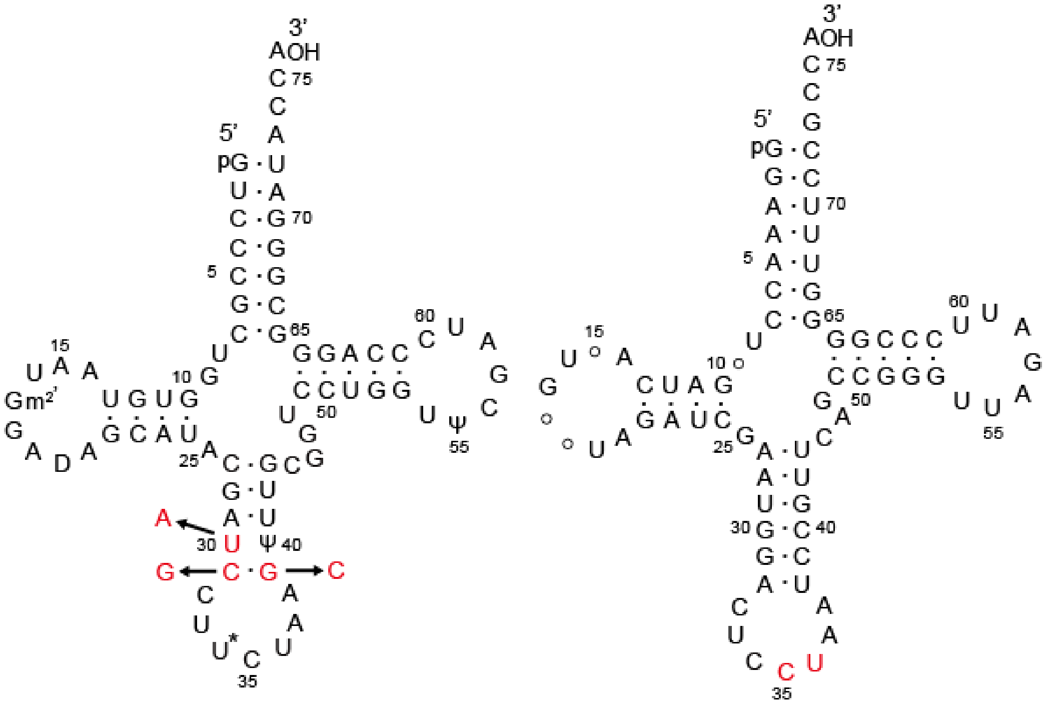

Figure S3. The secondary structures of the variant of T4 tRNA$^{Arg}_{UCU}$ (tRNA$^{T4}_{UCU}$) (left) and *Methanosarcina mazei* tRNA$^{Pyl}_{CCU}$ (right). The base substitutions are shown in red.

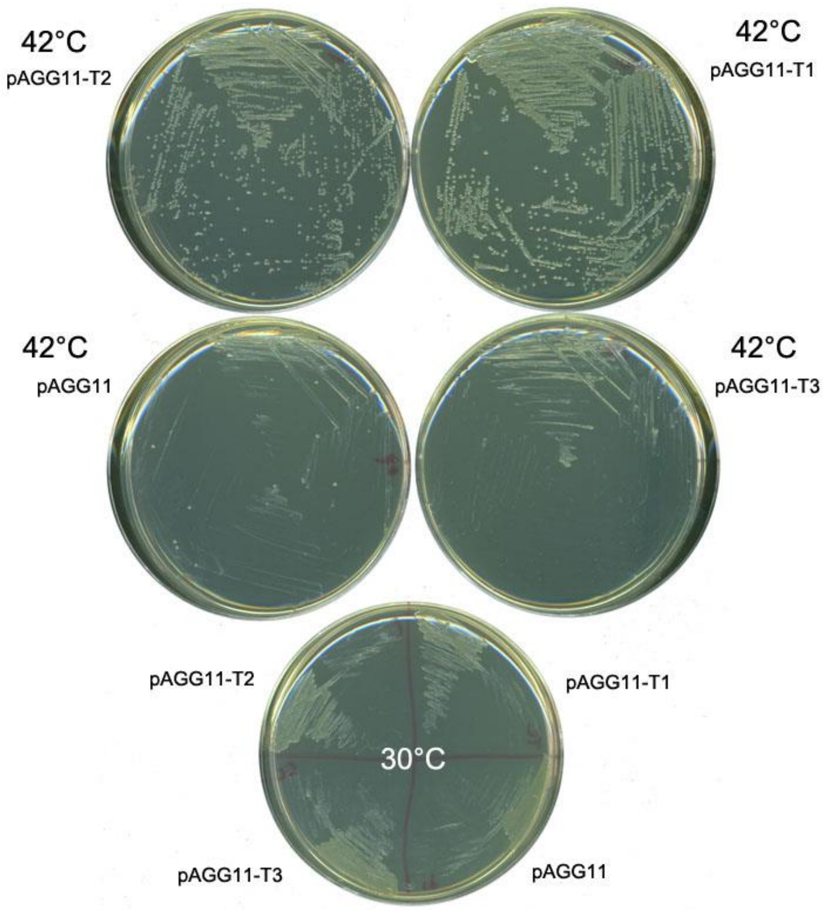

Figure S4. The complementation of the temperature-sensitivity of AGG-27.2 by expressing tRNA$^{T4}_{UCU}$ from various promoters. AGG-27.2 was transformed with pAGG11, pAGG11-T1, pAGG11-T2, and pAGG11-T3. The transformed cells were inoculated on LB agar plates and incubated at 30°C or 42°C for two days.

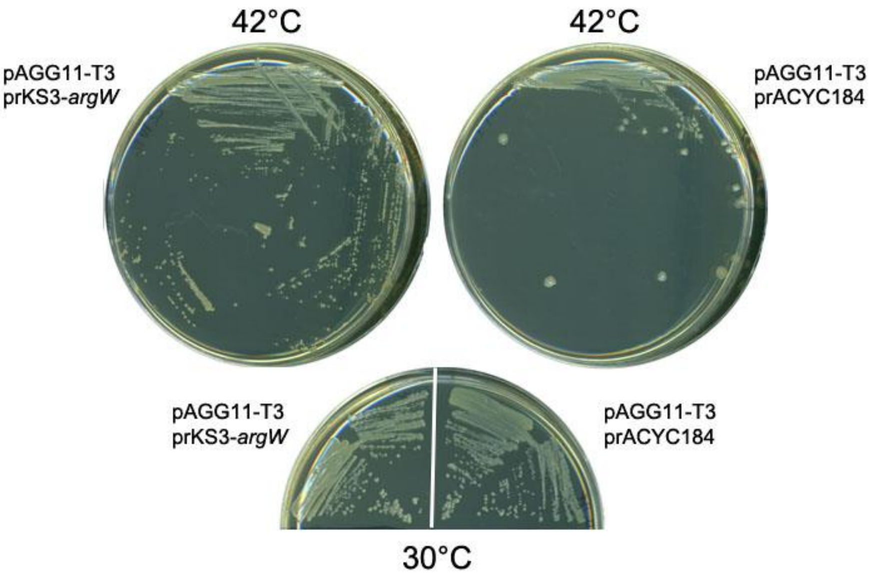

Figure S5. The complementation of the temperature sensitivity of AGG-27.2 by co-expressing $tRNA^{T4}_{UCU}$ and $tRNA^{Arg5}$. AGG-27.2 cells were transformed with pAGG11-T3 and a control plasmid (prACYC184), or with pAGG11-T3 and prKS3-*argW*. The transformants were inoculated on LB agar plates and incubated at 30°C and 42°C for two days.

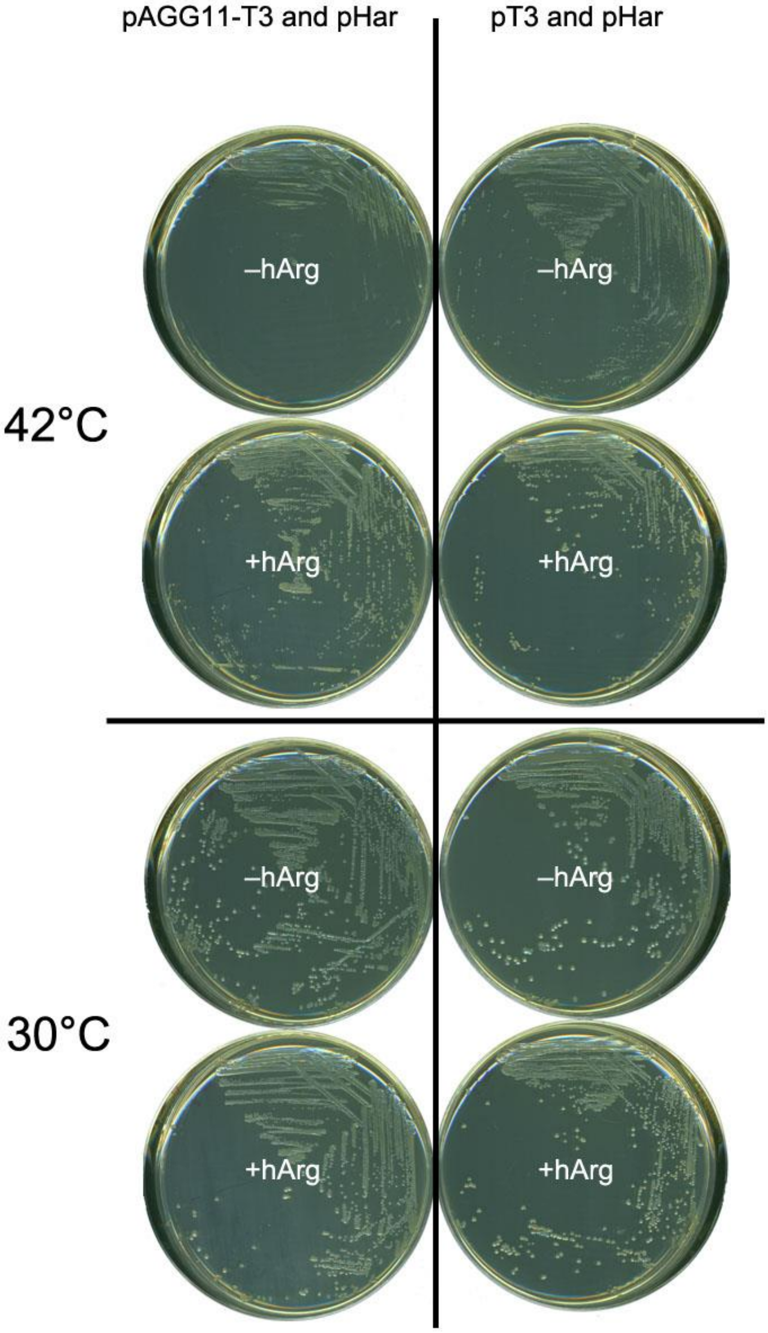

Figure S6. The complementation of the temperature-sensitivity of AGG-27.2 by reassigning AGG to L-homoarginine. The AGG-27.2 cells were transformed with pAGG11-T3 and pHar (the left column of the plates), and with pT3 and pHar (the right column). The transformants were inoculated on LB agar plates with or without L-homoarginine (hArg), and incubated at 30°C and 42°C for two days.

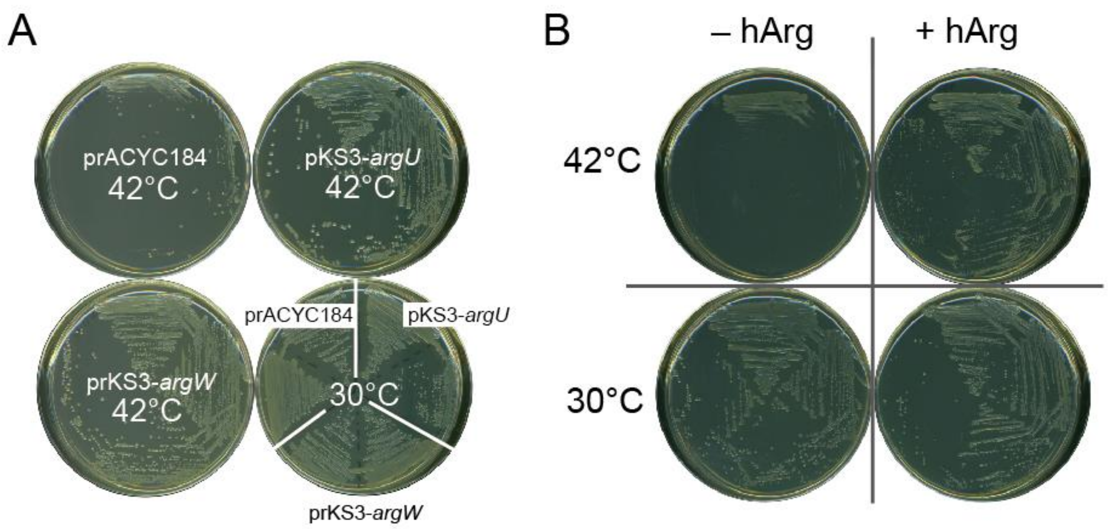

Figure S7. (*A*) The complementation of the temperature-sensitivity of AGG-27.3 by expressing $tRNA^{Arg5}$ or $tRNA^{Arg4}$. The AGG-27.3 cells transformed with prKS3-*argW*, pKS3-*argU*, or a control plasmid (prACYC184) were inoculated on LB agar plates, and incubated overnight at 30°C and 42°C. *(B)* The complementation of the temperature-sensitivity of AGG-27.3 by reassigning AGG to L-homoarginine (hArg). The AGG-27.3 cells transformed with pHar were inoculated on LB agar plates with or without hArg, and incubated for one day at 30°C and 42°C.

A

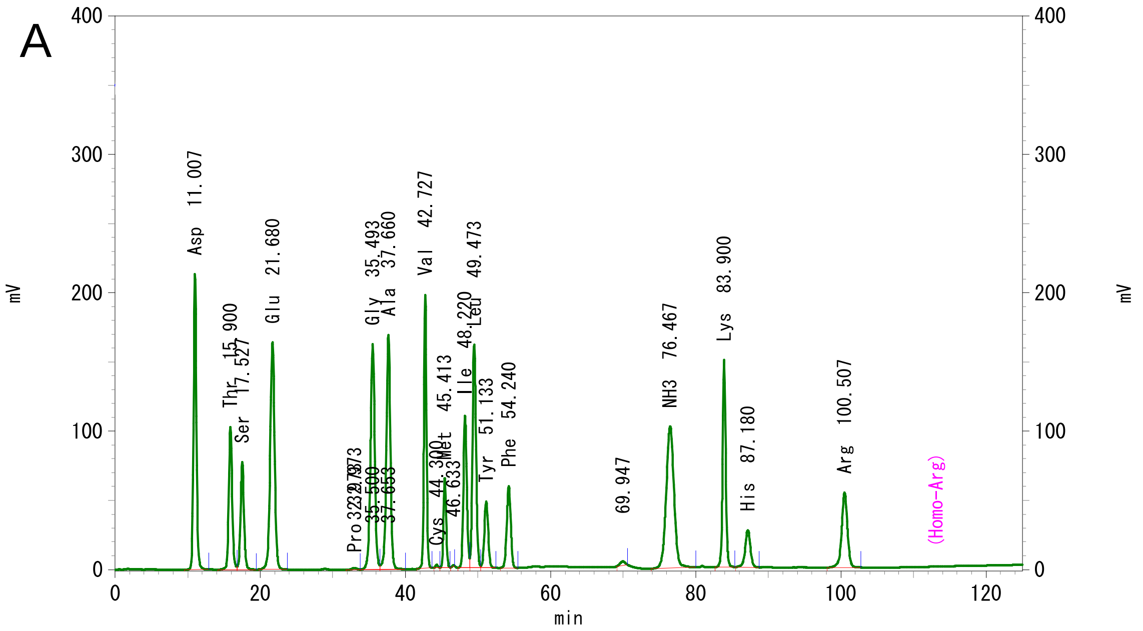

B

No.	retention time (min)	component	peak height	peak area	concentration (nmol)	weight (ng)	resolution (JP)
1	11.007	Asp	853822	23856878	11.89	1582.621	0
2	15.9	Thr	411786	13358009	6.248	744.163	6.41
3	17.527	Ser	311221	10217511	4.773	501.612	1.976
4	21.68	Glu	656144	27902984	13.26	1950.496	4.338
6	35.493	Gly	651343	27139868	12.114	909.435	2.283
7	37.66	Ala	677610	26581433	11.926	1062.461	2.118
8	42.727	Val	790939	19274966	8.805	1031.039	6.276
9	44.3	Cys	9236	200706	0.087	20.994	2.622
10	45.413	Met	257727	6628928	2.968	442.786	1.783
12	48.22	Ile	440001	14510555	6.71	880.348	2.33
13	49.473	Leu	644347	22166544	10.109	1326.315	1.419
14	51.133	Tyr	190684	7068568	3.257	590.095	1.774
15	54.24	Phe	236497	9019919	4.274	706.108	3.155
17	76.467	NH3	409178	30626356	20.215	344.259	3.971
18	83.9	Lys	598351	17868480	7.555	1104.568	5.517
19	87.18	His	106702	5300304	2.469	383.149	3.243
20	100.507	Arg	215985	11945208	5.686	990.495	10.017
		Homo-Arg			0.000BDL	0	

Figure S8. The quantitative analysis of the amino-acid composition of the soluble proteins from B-95.ΔA. (*A*) The profile of the chromatography separating the standard amino acids and L-homoarginine. Proline was not detected in this chromatography. (*B*) The list tabulates the data for each amino acid.

A

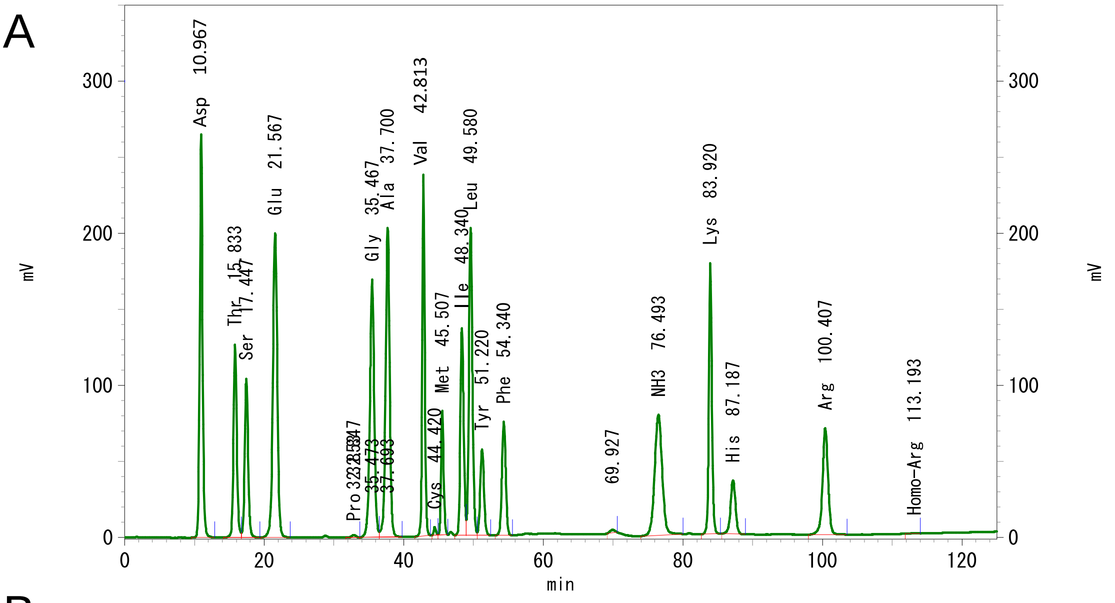

B

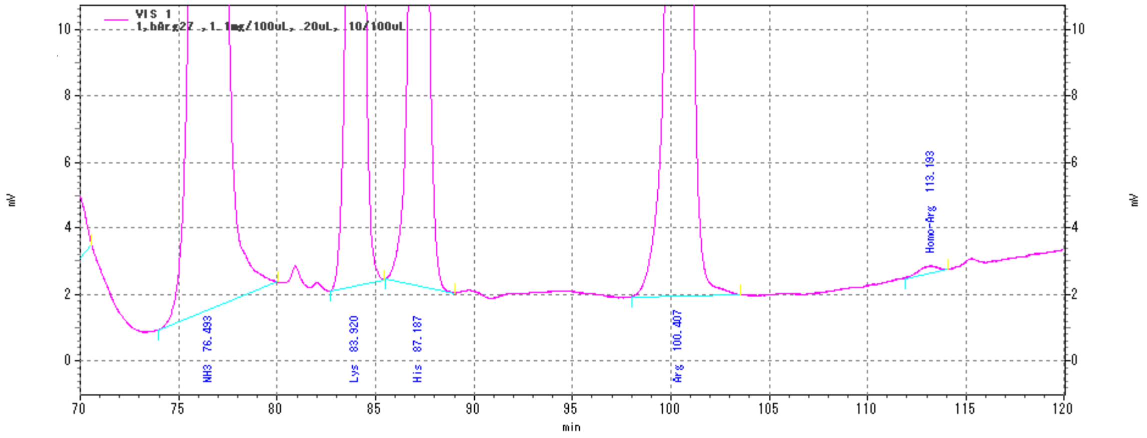

C

No.	retention time (min)	component	peak height	peak area	concentration (nmol)	weight (ng)	resolution (JP)
1	10.967	Asp	1060901	30142802	15.048	2002.894	0
2	15.833	Thr	507310	16757015	7.904	941.363	6.329
3	17.447	Ser	418016	13975661	6.546	688.011	1.933
4	21.567	Glu	800110	35144455	16.757	2464.89	4.203
6	35.467	Gly	677960	29643444	13.204	991.208	2.324
7	37.7	Ala	813298	33065155	14.96	1332.773	2.095
8	42.813	Val	950688	23747215	10.995	1287.536	6.189
9	44.42	Cys	21709	476637	0.211	50.82	2.701
10	45.507	Met	326700	8644403	3.801	567.124	1.742
11	48.34	Ile	544391	18320364	8.621	1131.127	3.609
12	49.58	Leu	808108	28444560	12.939	1697.6	1.374
13	51.22	Tyr	226074	8436057	3.885	703.926	1.73
14	54.34	Phe	298253	11313302	5.368	886.789	3.179
16	76.493	NH3	316607	23903336	15.886	270.545	3.959
17	83.92	Lys	713232	21534296	9.088	1328.639	5.487
18	87.187	His	140924	7032191	3.249	504.252	3.217
19	100.407	Arg	279171	15530771	7.392	1287.714	9.915
20	113.193	Homo-Arg	851	59356	0.033	6.241	7.977

Figure S9. The quantitative analysis of the amino-acid composition of the soluble proteins from AGG-27.3/Har. (*A*) The profile of the chromatography separating the standard amino acids and L-homoarginine. Proline was not detected in this chromatography. (*B*) A magnified area encompassing the L-homoarginine peak at 113 min. (*C*) The list tabulates the data for each amino acid.

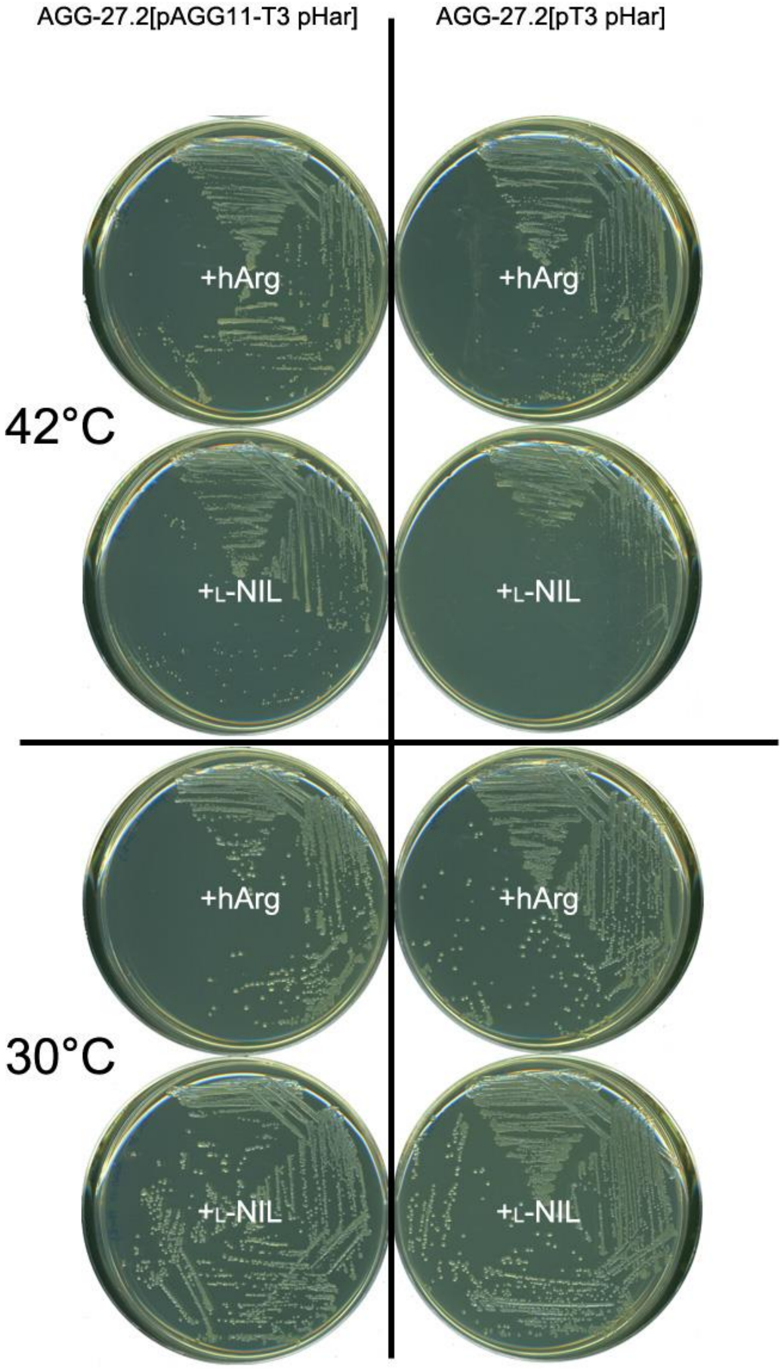

Figure S10. The temperature-sensitivity examined for the AGG-27.2[pAGG11-T3 pHar] and AGG-27.2[pT3 pHar] cells in the presence of L-homoarginine (hArg) and L-NIL in the growth media. The cells were incubated on LB agar plates containing the designated amino acids for 2 days at 30°C and 42°C.

Table S1. The base sequences of the tRNA$^{T4}_{UCU}$ gene including the indicated promoters and the *rrnC* terminator.

P*tyrT*-core-tRNA$^{T4}_{UCU}$-T*rrnC* (T1):

TTCTCAACGTAACACTTTACAGCGGCGCGTCATTTGATATGATGCGCCCCGCTTCCCGAT
AAGGGAGCAGGCCAGTAAAAAGGATCCATCAGACGCATTACGTCCCGCTGGTGTAATG
GATAGCATACGAAGCTTCTAACTTTGCGGTCCTGGTTCGATCCCAGGGCGGGATACCAA
ATTTATCACAGATTGGAAATTTTTGATCCTTAGCGAAAGCTAAGGATTTTTTTTATCGCG
ACGCGAGGCTGGATGGCCTTCCCCATTATGATTCTTCTCGCTTCCGGCGGCATCGGGAT
GCCCGCGTTGCAGGCCATGCTGTCCAGGCAGGTAGATGACGACCATCAGGGACAGCT

P*selC*-tRNA$^{T4}_{UCU}$-T*rrnC* (T2):

GCGAATATTCCGATATCTGGTTATTAATTTATGATTCTTGTTTTATGTGATCGTGGTAGC
GTTAATTCCGCTCATATATCATTGTAAAATATGGGTTTTATATGAACTATAATGCTTTCG
TGATAATACGCTGCGTGTATTAGGCGGAAAAAACTGATCTGGGGGATGTAGAAACTCA
AGGAAGTAGCTATAATGCGCCCCGCTTCCCGATAAGGGAGCAGGCCAGTAAAAAGGAT
CCATCAGACGCATTACGTCCCGCTGGTGTAATGGATAGCATACGAAGCTTCTAACTTTG
CGGTCCTGGTTCGATCCCAGGGCGGGATACCAAATTTATCACAGATTGGAAATTTTTGA
TCCTTAGCGAAAGCTAAGGATTTTTTTTATCGCGACGCGAGGCTGGATGGCCTTCCCCA
TTATGATTCTTCTCGCTTCCGGCGGCATCGGGATGCCCGCGTTGCAGGCCATGCTGTCCA
GGCAGGTAGATGACGACCATCAGGGACAGCT

P*selC*-core-tRNA$^{T4}_{UCU}$-T*rrnC* (T3):

GATCTGGGGGATGTAGAAACTCAAGGAAGTAGCTATAATGCGCCCCGCTTCCCGATAA
GGGAGCAGGCCAGTAAAAAGGATCCATCAGACGCATTACGTCCCGCTGGTGTAATGGA
TAGCATACGAAGCTTCTAACTTTGCGGTCCTGGTTCGATCCCAGGGCGGGATACCAAAT
TTATCACAGATTGGAAATTTTTGATCCTTAGCGAAAGCTAAGGATTTTTTTTATCGCGAC
GCGAGGCTGGATGGCCTTCCCCATTATGATTCTTCTCGCTTCCGGCGGCATCGGGATGC
CCGCGTTGCAGGCCATGCTGTCCAGGCAGGTAGATGACGACCATCAGGGACAGCT

Table S2. The oligonucleotides used for genome engineering. The lowercase letters represent the phosphorothioate parts within the oligonucleotides.

pheT	ttcaGCTTTTGTAAGCGCCATAGGTTCAATCCCTTAGTCTCGCAATGATGCCTGGAATCGCTCTTTTAATGCCTC
dnaC	gccgATATTATGTCGGCGATGAAAGATACCTTCCGCAATAGCGGTACCAGCGAAGAACAACTGCTTAACGATCTG
yejM	cccgCTGACCTTTATCGTCGGCTCCCAGCGCCTGATGCGCTTTTTGTCCGTCATTCTGGCAACGGCGGGAATGAC
lexA	tgatCACGGATGAGATCAAACACCTCTTGCTGTCTCGCAGTCAACGCTTTCATTCCGCCCCCTGGGTGTATATAC
coaE	tttgATAATCATGATGAATTCCCGGGAATATAAATTATGCGCTATATAGTTGCCTTAACGGGAGGCATTGGCAGT
trmD (for AGA)	ttcaGCTTTTGTAAGCGCCATAGGTTCAATCCCTTAGTCTCGCAATGATGCCTGGAATCGCTCTTTTAATGCCTC
trmD	ttgcTGTTGTGCGTGTTCCGTTTTGAACTCCGCCAAAAGTCGGGCTTGCTCTTCAGTCAGAGCCAGGTTTTCCAG
hemG	aggtAGGAGGCAATCTCGCGCGTTTGTCCGTCCCGAGTACTAAAAAGAATTAATGTTTTCACGTATTACTCCATC
ribF	gctgATGAAATTTTGCGCGGTTAACGCCGCAAAGCGACGATCAAAACGCACGCACAGCACGTAATCAACGCCACA
asd	catcAGGCTTACGGTACAGTTACCGCCAACAAAAGTCTTCACGCCATTATTTAATCCGTCGGTAATGACGTCCTG
lpxC	accgACACCCGTCGCCTGAACGATACGTTTCAAAGTACGCTGCTTGATCATCGTATTATCTCGCCAAATTACCTA
folC	actaACCGATTCTGAAACTTACTTGCCACTTAACCTCCTGAGCGACGCGCGTCAATCACTTCCATGACATGTGCG
secE	gcgcTTTTTAGGAGCTTCAGACATCTCAGAACCTCAAAACCGCAGGCCAGTGATAAAGGATACCAGGCGAACCAG
dnaT (for AGA)	gcgaACCTGACAGCCAAATTCCACCAGGATTCCGTGGTTAAGAGGGTAACGATGAAAAACGTTGGCGACCTGATG
holB (for AGA)	ttttCGAAATCAGGTCGTAACCATGGATACCATCGCATTAATCCAACTCCTTCACCCAGTGGGTCACGGTAGTGC
yceQ	tggaATAATGAGGCCGTTTCCGTGTCCATTCGAGTAAGGACTAAAAATTTTACGGAATAACCCATTTTGCCCGAC
ftsI	gttaCCATTAGCCCGTAGCCGAAAGAGAAAGTCGCACGTTCAATGTCAGACCACCGTTGTTTTTGAGGATATAAG

lptC<br>(for AGA)	cagcAAGAACGCCGAGCTGATTGAAAAGGTACGGACCAGTTATGAAATTCAAAA<br>CAAACAAACTCAGCCTTAATC
ftsL	atcaACATGCTGCATTTGCAGCTTTTCCGTCGCAATACGCTCAACCCGGCTATGGT<br>CGCCGAGCGCATTCTCTTC
dnaG	gcagGCAGCGGCCCCAGCCAGATCGAGCGCCACCAACGTCAGACCCTTTATCAGT<br>TGATGGACGGTCTGAATACG
minE<br>(for repair)	caaaTTGATCCTGAGATGGTAACCGTACAATTGGAACAGAAAGATGGCGATATTT<br>CTATTCTTGAGCTGAACGTG
cca<br>(for AGA)	tctgCGCGTGCCAAATGAAATTCGCGATTTAGCGCGCTTAGTGGCTGAGTTTCACG<br>ATCTCATCCACACCTTCCC
lptF	agatTCGCCGGAATATCGCCGTCAACCGCTGCCCCCAGAATACGCACTAACTTTTG<br>ACAGAAGAAGATCAAAAGC
ftsB	caccAGACGATAAAAAGTTTCGCCCGGCCGGGTCATCGACAGTTCATTACGCGCA<br>CGCTCTTCGAGCGCCTCCTG
glyQ	taatCCTGTAAGGTCAGGATCAAGCCCTGGAACGTACGCGTATCAAACTTTTGCAT<br>ATTATTTCGTGCTGGATAC

Table S3. The primer pairs used for colony-direct PCR. The numbers after the gene names indicate the expected lengths (bp) of the PCR products.

Gene, size(bp)	Forward primer	Reverse primer
pheT, 410	GGTTGTTCATCCTGAACTGG	GGTTCAATCCCTTAGTCTCG
dnaC, 290	GATGAAAGATACCTTCCGC	CTACGGTAGCTATCCCAGTTG
yejM, 300	AAGCGCACTAAGGGAAACAG	AATGACGGACAAAAAGCG
lexA, 400	CGTAGCGGTATTGTTGGCG	TCTTGCTGTCTCGCAGTC
coaE, 259	CGGGAATATAAATTATGCGC	TTTTTCTCTTCCGGGTTGGCG
trmD (for AGA), 350	AATTGATTCTGGTGTGCGGTC	AGAAGTTCAGGACGACGC
trmD, 400	AATTGATTCTGGTGTGCGGTC	TTTGAACTCCGCCAAAAG
hemG, 200	CGTGAAAACATTAATTCTTTTTAGTACTC	GGAACGCTGAATGGTAGTGAC
ribF, 200	CGTTTTGATCGTCGCTTT	CCTTCGCAAAAAGTTTGCGTAC
asd, 400	AAAACGCTTATGAAAAATGTTGG	CCGCCAACAAAAGTCTTC
lpxC, 600	GATCAAGCAGCGTACTTTG	GATATTCGATATCACGCATGAAAC
folC, 500	CGTCGCTCAGGAGGTTAAG	TCACTTTATCGGCATTTTTCAG
secE, 300	CAACTACCTTTATCGCGACATTATG	ATCTCAGAACCTCAAAACCG
dnaT, 506	CCTGGTACACGATCACCAAAC	CGTTACCCTCTTAACCACG
holB, 200	TGAAGGAGTTGGATTAATGC	AGTGACCGCAACTTTTGTGAC
yceQ, 259	CAACCTCACGGTTATCGTCAG	CGTGTCCATTCGAGTAAGG
ftsI, 400	TGACATTGAACGTGCGACT	GTAGTATTTACCCGCCTGCG
lptC, 400	TTGAAAAGGTACGGACCAG	CATTACCCGTCAGAACGAC
ftsL, 300	GTGACAGAAGCTCTAAGCAAAG	TTCCGTCGCAATACGCTCA
dnaG, 326	CATTAATGATCTGCTGGCAC	GATAAAGGGTCTGACGTTGG
minE (for repair), 200	GTAACCGTACAATTGGAACAG	GCAATGAGGAGTATCAGCAAG
cca (for AGA), 300	AAATTCGCGATTTAGCGC	TCAGCTCCTCGCGAATCTC
lptF, 497	GTTAGTGCGTATTCTGGGG	GTTAAATGTCCGGAATCGG
ftsB, 242	CGCGTAATGAACTGTCG	CGAGTGTTCAAGAATGGTTTG
glyQ, 510	atATGCAAAAGTTTGATACGC	GACGTTCCAGACCGTAGGTG

Table S4. Numbers of AGA and AGG codons included in the essential open reading frames (ORFs) of the *E. coli* K-12, BL21(DE3), and AGG-27.1 strains.

	K-12		BL21(DE3)		AGG-27.1	
name	AGA	AGG	AGA	AGG	AGA	AGG
ileS	0	0	0	0	0	0
lspA	1	0	0	0	0	0
ispH	0	0	0	0	0	0
dapB	2	0	2	0	2	0
folA	0	0	0	0	0	0
imp	2	0	2	0	2	0
murE	0	0	0	0	0	0
murF	0	0	0	0	0	0
mraY	0	0	0	0	0	0
murD	0	0	0	0	0	0
ftsW	0	0	0	0	0	0
murG	0	0	0	0	0	0
murC	0	0	0	0	0	0
ftsQ	2	0	2	0	2	0
ftsA	1	0	1	0	1	0
ftsZ	0	0	0	0	0	0
yacA	0	0	0	0	0	0
secA	0	0	0	0	0	0
yadF	0	0	0	0	0	0
folK	1	0	1	0	1	0
hemL	0	0	0	0	0	0
yadR	0	0	0	0	0	0
dapD	0	0	0	0	0	0
map	0	0	0	0	0	0
rpsB	0	0	0	0	0	0
tsf	0	0	0	0	0	0
pyrH	0	0	0	0	0	0
frr	1	0	1	0	1	0
dxr	1	0	1	0	1	0
yaeT	0	0	0	0	0	0
lpxD	0	0	0	0	0	0
fabZ	0	0	0	0	0	0
lpxA	0	0	0	0	0	0
lpxB	2	0	2	0	2	0
accA	0	0	0	0	0	0
secD	0	0	0	0	0	0
secF	0	0	0	0	0	0
ribD	0	0	0	0	0	0
ribE	0	0	0	0	0	0
nusB	0	0	0	0	0	0
thiL	1	0	1	0	1	0
dxs	0	0	0	0	0	0
ispA	1	0	1	0	1	0
dnaX	1	0	1	0	1	0
adk	0	0	0	0	0	0
hemH	1	0	1	0	1	0
lpxH	0	0	0	0	0	0
cysS	0	0	0	0	0	0
folD	0	0	0	0	0	0
mrdA	1	0	1	0	1	0
nadD	0	0	0	0	0	0
holA	0	0	0	0	0	0
rlpB	0	0	0	0	0	0
leuS	0	0	0	0	0	0
lnt	0	0	0	0	0	0
glnS	0	0	0	0	0	0
fldA	0	0	0	0	0	0
infA	0	0	0	0	0	0
ftsK	0	0	0	0	0	0
lolA	0	0	0	0	0	0
serS	0	0	0	0	0	0
rpsA	0	0	0	0	0	0
msbA	0	0	0	0	0	0
lpxK	0	0	0	0	0	0
kdsB	0	0	0	0	0	0
mukF	2	0	2	0	2	0
mukE	0	0	0	0	0	0
mukB	0	0	0	0	0	0
fabA	0	0	0	0	0	0
mviN	1	0	1	0	1	0
rne	1	0	1	0	1	0
fabD	0	0	0	0	0	0
fabG	0	0	0	0	0	0
acpP	0	0	0	0	0	0
tmk	1	0	1	0	1	0
holB	2	0	2	0	1	0
lolC	0	0	0	0	0	0
lolD	0	0	0	0	0	0

lolE	1	0	1	0	1	0
ycfB	0	0	0	0	0	0
pth	0	0	0	0	0	0
prsA	0	0	0	0	0	0
lolB	0	0	0	0	0	0
hemA	0	0	0	0	0	0
prfA	0	0	0	0	0	0
hemK	1	0	0	0	0	0
kdsA	0	0	0	0	0	0
topA	0	0	0	0	0	0
ribA	0	0	0	0	0	0
fabI	0	0	0	0	0	0
tyrS	0	0	0	0	0	0
ribC	0	0	0	0	0	0
pheS	0	0	0	0	0	0
rplT	0	0	0	0	0	0
infC	1	0	1	0	1	0
thrS	1	0	1	0	1	0
nadE	1	0	1	0	1	0
gapA	0	0	0	0	0	0
yeaZ	0	0	0	0	0	0
aspS	0	0	0	0	0	0
argS	0	0	0	0	0	0
pgsA	0	0	0	0	0	0
folE	0	0	0	0	0	0
gyrA	1	0	1	0	1	0
nrdA	0	0	0	0	0	0
nrdB	0	0	0	0	0	0

accD	0	0	0	0	0	0
fabB	0	0	0	0	0	0
gltX	0	0	0	0	0	0
zipA	0	0	0	0	0	0
dapE	0	0	0	0	0	0
dapA	1	0	1	0	1	0
hda	1	0	1	0	1	0
der	0	0	0	0	0	0
hisS	0	0	0	0	0	0
ispG	1	0	1	0	1	0
suhB	0	0	0	0	0	0
acpS	0	0	0	0	0	0
era	0	0	0	0	0	0
lepB	0	0	0	0	0	0
yfiO	1	0	1	0	1	0
rplS	0	0	0	0	0	0
yfjA	1	0	1	0	1	0
rpsP	0	0	0	0	0	0
ffh	1	0	1	0	1	0
grpE	0	0	0	0	0	0
yfjB	0	0	0	0	0	0
csrA	0	0	0	0	0	0
alaS	0	0	0	0	0	0
ispF	0	0	0	0	0	0
ispD	0	0	0	0	0	0
eno	0	0	0	0	0	0
pyrG	0	0	0	0	0	0
lgt	0	0	0	0	0	0

fbaA	0	0	0	0	0	0
pgk	0	0	0	0	0	0
metK	0	0	0	0	0	0
yqgF	1	0	1	0	1	0
plsC	1	0	1	0	1	0
parC	0	0	0	0	0	0
parE	0	0	0	0	0	0
ribB	0	0	0	0	0	0
cca	3	0	3	0	2	0
ygiG	0	0	0	0	0	0
ygjD	0	0	0	0	0	0
rpoD	0	0	0	0	0	0
infB	0	0	0	0	0	0
nusA	0	0	0	0	0	0
mrsA	0	0	0	0	0	0
ftsH	0	0	0	0	0	0
obgE	0	0	0	0	0	0
rpmA	0	0	0	0	0	0
rplU	0	0	0	0	0	0
murA	0	0	0	0	0	0
yrbI	0	0	0	0	0	0
lptC	3	0	3	0	2	0
yhbN	0	0	0	0	0	0
yhbG	1	0	1	0	1	0
rpsI	0	0	0	0	0	0
rplM	0	0	0	0	0	0
degS	0	0	0	0	0	0
mreD	1	0	1	0	1	0

mreB	0	0	0	0	0	0
accB	0	0	0	0	0	0
accC	0	0	0	0	0	0
yrdC	1	0	1	0	1	0
def	0	0	0	0	0	0
fmt	0	0	0	0	0	0
rplQ	0	0	0	0	0	0
rpoA	1	0	1	0	1	0
rpsD	1	0	1	0	1	0
rpsK	1	0	1	0	1	0
rpsM	0	0	0	0	0	0
secY	1	0	1	0	1	0
rplO	0	0	0	0	0	0
rpmD	0	0	0	0	0	0
rpsE	0	0	0	0	0	0
rplR	0	0	0	0	0	0
rplF	0	0	0	0	0	0
rpsH	0	0	0	0	0	0
rpsN	0	0	0	0	0	0
rplE	0	0	0	0	0	0
rplX	1	0	1	0	1	0
rplN	0	0	0	0	0	0
rpsQ	0	0	0	0	0	0
rpmC	0	0	0	0	0	0
rplP	0	0	0	0	0	0
rpsC	0	0	0	0	0	0
rplV	0	0	0	0	0	0
rpsS	0	0	0	0	0	0

rplB	0	0	0	0	0	0
rplW	0	0	0	0	0	0
rplD	0	0	0	0	0	0
rplC	0	0	0	0	0	0
rpsJ	1	0	1	0	1	0
fusA	0	0	0	0	0	0
rpsG	0	0	0	0	0	0
rpsL	0	0	0	0	0	0
trpS	0	0	0	0	0	0
yrfF	1	0	1	0	1	0
rpoH	0	0	0	0	0	0
ftsX	0	0	0	0	0	0
ftsE	1	0	1	0	1	0
ftsY	0	0	0	0	0	0
glyS	0	0	0	0	0	0
gpsA	1	0	1	0	1	0
kdtA	0	0	0	0	0	0
coaD	0	0	0	0	0	0
rpmB	0	0	0	0	0	0
dfp	0	0	0	0	0	0
dut	0	0	0	0	0	0
gmk	1	0	1	0	1	0
spoT	0	0	0	0	0	0
gyrB	0	0	0	0	0	0
dnaN	1	0	1	0	1	0
dnaA	1	0	1	0	1	0
rpmH	0	0	0	0	0	0
yidC	0	0	0	0	0	0

glmS	0	0	0	0	0	0
glmU	0	0	0	0	0	0
rho	0	0	0	0	0	0
hemD	0	0	0	0	0	0
hemC	1	0	1	0	1	0
yihA	0	0	0	0	0	0
ftsN	1	0	1	0	1	0
priA	0	0	0	0	0	0
murI	2	0	1	0	1	0
murB	1	0	1	0	1	0
birA	0	0	0	0	0	0
coaA	1	0	1	0	1	0
nusG	0	0	0	0	0	0
rplJ	0	0	0	0	0	0
rplL	0	0	0	0	0	0
rpoB	0	0	0	0	0	0
rpoC	0	0	0	0	0	0
ubiA	0	0	0	0	0	0
plsB	1	0	0	0	0	0
dnaB	0	0	0	0	0	0
ssb	1	0	1	0	1	0
groES	0	0	0	0	0	0
groEL	0	0	0	0	0	0
efp	0	0	0	0	0	0
psd	0	0	0	0	0	0
yjeQ	0	0	0	0	0	0
orn	0	0	0	0	0	0
yjeE	0	0	0	0	0	0

rpsR	0	0	0	0	0	0
ppa	0	0	0	0	0	0
valS	0	0	0	0	0	0
yjgQ	1	0	1	0	1	0
dnaT	3	0	3	0	2	0
ribF	1	1	0	1	0	0
ftsL	1	1	1	1	1	0
ftsI	1	1	1	1	1	0
lpxC	0	1	0	1	0	0
coaE	0	1	0	1	0	0
cdsA	0	1	0	1	0	1
tilS	1	1	1	0	1	0
proS	0	1	0	0	0	0
hemB	0	0	0	0	0	0
mrdB	1	1	1	1	1	0
asnS	0	1	0	1	0	0
ispE	2	1	2	1	2	0
pheT	0	1	0	1	0	0
metG	0	1	0	0	0	0
folC	1	1	1	1	0	0
ligA	0	1	0	1	0	0
pssA	0	1	0	1	0	0
trmD	2	1	2	1	0	0
ftsB	0	1	0	1	0	0
dnaG	1	1	1	1	1	0
ispB	0	1	0	1	0	0
mreC	0	1	0	1	0	0
asd	0	1	0	1	0	0

glyQ	0	1	0	1	0	0
rnpA	0	1	0	1	0	1
hemG	0	1	0	1	0	0
secE	0	1	0	1	0	0
lexA	0	1	0	1	1	0
lptF	1	1	1	1	1	0
dnaC	0	1	0	1	0	0
ispU	0	2	0	2	0	2
rseP	0	2	0	2	0	2
yceQ	0	1	0	1	0	0
yejM	1	2	1	2	1	0
prfB	0	1	0	1	0	1
dnaE	0	4	0	4	0	4

Table S5. The *m/z* values of the fragments from the AHHHHHH*L peptide (the asterisk indicates L-homoarginine) observed in the MS/MS spectrum of Figure 1D.

m/z (calculated)	*m/z* (observed)	b		y	*m/z* (calculated)	*m/z* (observed)
	---	1	A	9	---	
209.1	209.1	2	H	8	1124.6	1124.6
346.1	346.2	3	H	7	987.5	987.5
483.2	483.2	4	H	6	850.5	850.3
620.2	620.3	5	H	5	713.4	713.3
757.3	757.3	6	H	4	576.3	576.2
894.5	894.4	7	H	3	439.3	439.2
1064.5	1064.5	8	hArg	2	302.2	302.2
	---	9	L	1	132.1	

Table S6. The 7 synonymous AGG replacements in B-95.ΔA. These replacements were accomplished by oligonucleotide-mediated recombination. The changed bases are shown in red, except for those introduced by the C-to-C mismatches, which are shown in green. The AGG codons are underlined.

mreC	AGG to CGG
before	TGCTAATCGCTCTCCACAAAGGGCTACGCCGCCGCAAAGT
after	TGCTAATCGTAGCCCGCAACGGGCTACGCCGCCGCAAAGT
ligA	AGG to CGT
before	TCCCGACGCTGAATACGACAGGCTGATGCGCGAACTGCGC
after	TCCCGACGCTGAATACGATCGTTTAATGCGCGAACTGCGC
mrdB	AGG to CGC
before	AATGTCAATCCACACCCACAGGAAAATGTTGTCGAAAAGC
after	AATGTCAATCCACACCCACCGCAAAATGTTGTCGAAAAGC
note	P*rlpA* -10 box is written in enclosed characters.
ispE	AGG to CGC
before	CCCGCGCAATACGCCAAAAAGGTCAATAGAAACGTTGCTA
after	CCCGCGCAATACGCCAAAACGCTCAATAGAAACGTTGCTA
asnS	AGG to CGC
before	CATTCTCGAAAACTGCGGCAGGAAGTTTGAAAACCCGGTT
after	CATTCTCGAAAACTGCGGCCGCAAGTTTGAAAACCCGGTT
ispB	AGG to CGC
before	GGATGAATCAGATATGCGCAGGGGTAAAGCTACCGCCAAC
after	GGATGAATCAGATATGCGCCGCGGTAAAGCTACCGCCAAC
pssA	AGG to CGC
before	CGCGTTGTATGAAGCTAAAAGGCAGCGTCCGGAACTGGAT
after	CGCGTTGTATGAAGCTAAACGCCAGCGTCCGGAACTGGAT

Table S7. The sequence modifications to the B-95.ΔA chromosome made in this study. The base changes were performed by oligonucleotide-mediated recombination. The original and modified sequences are designated as "before" and "after", respectively, on the left. The modified genes are listed in the order of the actual engineering steps. The changed bases are shown in red, except for those introduced by the C-to-C mismatches, which are shown in green. The engineered codons are underlined.

pheT	AGG to CGA by 8-nucleotide insertion
before	TCCAGGCATCATTGAGGGATTGAACCTatggcg
after	TCCAGGCATCATTGCGAGACTAAGGGATTGAACCTatggcg
note	The *infA* ORF is written in lowercase letters.
dnaC	AGG to CGC
before	GGCGATGAAAGATACCTTCAGGAATAGCGGTACCAGCGAA
after	GGCGATGAAAGATACCTTCCGCAATAGCGGTACCAGCGAA
yejM	AGG/AGG to CGC/CGC
before	TCGTCGGCTCCCAGAGGCTGATGAGGTTTTTGTCCGTCAT
after	TCGTCGGCTCCCAGCGCCTGATGCGCTTTTTGTCCGTCAT
lexA	AGG to AGA
before	AATGAAAGCGTTAACGGCCAGGCAACAAGAGGTGTTTGAT
after	AATGAAAGCGTTGACTGCGAGACAGCAAGAGGTGTTTGAT
coaE	AGG to CGC
before	CCCGGGAATATAAATTATGAGGTATATAGTTGCCTTAACG
after	CCCGGGAATATAAATTATGCGCTATATAGTTGCCTTAACG
trmD	AGAAGA to CGTCGT
before	TGGGCCGTACCTGGCTTAGAAGACCTGAACTTCTGGAAAA
after	TGGGCCGTACCTGGTTGCGTCGTCCTGAACTTCTGGAAAA
trmD	AGG to CGA
before	TCTGACTGAAGAGCAAGCAAGGTTGCTGGCGGAGTTCAAA
after	TCTGACTGAAGAGCAAGCCCGACTTTGGCGGAGTTCAAA

hemG AGG to CGG

before ATTAATTCTTTTCTCAACAAGGGACGGACAAACGCGCGAG

after ATTAATTCTTTTTAGTACTCGGGACGGACAAACGCGCGAG

ribF AGG to CGT

before GCTGTGCGTGCGTTTCGACAGGCGTTTCGCGGCGTTAACC

after GCTGTGCGTGCGTTTTGATCGTCGCTTTGCGGCGTTAACC

asd ATCAGG to GTGAAG

before CGGATTAAATAATGGCATCAGGACTTTTGTTGGCGGTAAC

after CGGATTAAATAATGGCGTGAAGACTTTTGTTGGCGGTAAC

note Ile127-Arg128 was changed to Val127-Lys128 which is found in other *asd* genes.

lpxC AGG to CGT

before TAATACGATGATCAAACAAAGGACACTTAAACGTATCGTT

after TAATACGATGATCAAGCAGCGTACTTTGAAACGTATCGTT

folC AGGAGA to CGTCGC by 8-nucleotide insertion

before TGATTGACGCGAGGAGAAGCGgtggcaagtaa

after TGATTGACGCGCGTCGCTCAGGAGGTTAAgtggcaagtaa

note Since the 8-base insertion (in red, left) shifted the coding frame, “AAGCG” at the 3’ end of *folC* was changed to “GTTAA” to create the TAA stop signal (underlined) for premature termination.

note The *dedD* ORF is written in lowercase letters.

secE AGG to CGG by 8-nucleotide insertion

before TTTATCACTGGCCTGAGGTTCTGAGatgtctg

after TTTATCACTGGCCTGCGGTTTTGAGGTTCTGAGatgtctg

note The *nusG* ORF is written in lowercase letters.

dnaT AGA to CGT by 8-nucleotide insertion

before ATTCCACCAGGATTCAGAGGGTAACGatgaaa

after ATTCCACCAGGATTCCGTGGTTAAGAGGGTAACGatgaaa

note The *dnaC* ORF is written in lowercase letters.

holB	AGA to CGA
before	tgaaggagttggacgcatgaGATGGTATCCATGGTTACGA
after	tgaaggagttggattaaTGCGATGGTATCCATGGTTACGA
note	The *tmk* ORF is written in lowercase letters.
note	Since the AGA-to-CGA change eliminated the original stop codon (TGA) of *tmk*, a TAA stop signal (the first 2 bases shown in red, left) was created immediately upstream of the TGA.
yceQ	AGG to CGA
before	AAAATTTCTTGTTTTAACAAGGATGGACACGGAAACGGCC
after	AAAATTTTTAGTCCTTACTCGAATGGACACGGAAACGGCC
ftsI	AGG to CGT
before	ACGGTGGTCTGACATAGAGAGGGCCACCTTCTCTTTCGGC
after	ACGGTGGTCTGACATTGAACGTACGACTTTCTCTTTCGGC
note	An oligo-derived spontaneous mutation (A413T) was introduced.
lptC	AGA to CGG
before	GAGCTGATTGAAAAGGTTAGAACATCCTatgaaattcaaa
after	GAGCTGATTGAAAAGGTACGGACCAGTTatgaaattcaaa
note	The *lptA* ORF is written in lowercase letters.
note	The P*lptB* -35 box is enclosed.
ftsL	AGG to CGT
before	CGACCATAGCCGGGTGGAAAGGATCGCCACGGAAAAGCTG
after	CGACCATAGCCGGGTTGAGCGTATTGCGACGGAAAAGCTG
dnaG	AGG to CGT
before	CCAGATCGAGCGCCATCAGAGGCAAACGCTTTATCAGTTG
after	CCAGATCGAGCGCCACCCAACGTCAGACCCTTTATCAGTTG
minE	Correction of the off-target mutation in the *minE* gene
before	GGTAACCGTACAGCTTGAGCCAAAAGATGGCGATATTTCT
after	GGTAACCGTACAATTGGAACAGAAAGATGGCGATATTTCT
note	Pro at position 67 was corrected to be Gln.

cca	AGA to CGC
before	TGAAATTCGCGATTTAGCCAGACTGGTGGCTGAGTTTCAC
after	TGAAATTCGCGATTTAGCGCGCTTAGTGGCTGAGTTTCAC
lptF	AGG to CGT
before	CTTCTGTCAAAAGTTAGTGAGGATCCTCGGCGCAGCGGTT
after	CTTCTGTCAAAAGTTAGTGCGTATTCTGGGGGCAGCGGTT
ftsB	AGG to CGG
before	TAATGAACTCAGCATGACCAGGCCGGGCGAAACTTTTTAT
after	TAATGAACTGTCGATGACCCGGCCGGGCGAAACTTTTTAT
glyQ	AGG to CGT
before	TATGCAAAAGTTTGATACCAGGACCTTCCAGGGCTTGATC
after	TATGCAAAAGTTTGATACGCGTACGTTCCAGGGCTTGATC

Table S8. The off-target mutations occurring in AGG-27.1. These mutations were revealed by the whole-genome sequencing. The obtained sequences were compared with the standard genome sequence of BL21(DE3). The last three mutations are not present in the parent B-95.ΔA, and must have occurred during the engineering of AGG-27.1. The repeating DNA sequences, including rDNA, insertion elements, and *rhs* genes, were not completely mapped in this sequence analyses.

Annotation	Position	Type	Reference	Sequenced
acrB(I65I)	453255	Substitution	G	A
flgF(A19V)	1136454	Substitution	C	T
yebT(V66V)	1863432	Substitution	T	C
vioA(A338V)	2006172	Substitution	G	A
yicI(H304L)	3701104	Substitution	T	G
yjiPQ(L576L)	4478852	Substitution	T	A
cdaR(L52L)	185461	Substitution	G	A
upstream of *sulA*	1026026	Substitution	T	C
terminator of *pspF*	1353260	Substitution	G	A
dcp(ΔA)	1575919	Deletion	T	
yfbS(L39L)	2298490	Substitution	A	G
hemX(P389PAP)	3878170-1	Insertion		GGTGCA
tolQ(L130L)	734203	Substitution	C	T
yjhB(ΔT)	4415207	Deletion	T	
ftsI(A413T)	95454	Substitution	G	A
IS1 before *fepE*	578621-19	IS1 insertion		
yohGH(T19T)	2123535	Substitution	G	T
